# Genome Features of “Dark-Fly”, a *Drosophila* Line Reared Long-Term in a Dark Environment

**DOI:** 10.1371/journal.pone.0033288

**Published:** 2012-03-14

**Authors:** Minako Izutsu, Jun Zhou, Yuzo Sugiyama, Osamu Nishimura, Tomoyuki Aizu, Atsushi Toyoda, Asao Fujiyama, Kiyokazu Agata, Naoyuki Fuse

**Affiliations:** 1 Laboratory for Biodiversity, Global COE Program, Graduate School of Science, Kyoto University, Kyoto, Japan; 2 Laboratory for Molecular Developmental Biology, Graduate School of Science, Kyoto University, Kyoto, Japan; 3 Department of Organismic and Evolutionary Biology, Harvard University, Cambridge, Massachusetts, United States of America; 4 Comparative Genomics Laboratory, National Institute of Genetics, Mishima, Japan; Emory University School of Medicine, United States of America

## Abstract

Organisms are remarkably adapted to diverse environments by specialized metabolisms, morphology, or behaviors. To address the molecular mechanisms underlying environmental adaptation, we have utilized a *Drosophila melanogaster* line, termed “Dark-fly”, which has been maintained in constant dark conditions for 57 years (1400 generations). We found that Dark-fly exhibited higher fecundity in dark than in light conditions, indicating that Dark-fly possesses some traits advantageous in darkness. Using next-generation sequencing technology, we determined the whole genome sequence of Dark-fly and identified approximately 220,000 single nucleotide polymorphisms (SNPs) and 4,700 insertions or deletions (InDels) in the Dark-fly genome compared to the genome of the Oregon-R-S strain, a control strain. 1.8% of SNPs were classified as non-synonymous SNPs (nsSNPs: i.e., they alter the amino acid sequence of gene products). Among them, we detected 28 nonsense mutations (i.e., they produce a stop codon in the protein sequence) in the Dark-fly genome. These included genes encoding an olfactory receptor and a light receptor. We also searched runs of homozygosity (ROH) regions as putative regions selected during the population history, and found 21 ROH regions in the Dark-fly genome. We identified 241 genes carrying nsSNPs or InDels in the ROH regions. These include a cluster of alpha-esterase genes that are involved in detoxification processes. Furthermore, analysis of structural variants in the Dark-fly genome showed the deletion of a gene related to fatty acid metabolism. Our results revealed unique features of the Dark-fly genome and provided a list of potential candidate genes involved in environmental adaptation.

## Introduction

Organisms display traits beautifully adaptive for their environments. How organisms come to possess adaptive traits is a fundamental question for evolutionary biology. It is accepted that genomic alterations lead to diverse traits, and adaptive traits are then selected during evolutionary history. To understand the mechanisms of environmental adaptation, it is necessary to link genome to trait. Previous studies have identified genomic alterations causing evolved traits [1], for example, skin albinism of cavefish [2], wing spot gain of a *Drosophila* species [3], and pelvic loss of freshwater sticklebacks [4]. Those studies took mainly two approaches: “candidate gene studies” examined the genes most likely involved in the trait, while “quantitative trait loci studies” characterized the whole genome but evaluated major effects of a few genes. As a next step toward understanding the molecular evolution of adaptive traits, we need to view the whole genome sequence of the evolved organisms and to evaluate the effects of multiple genes. However, it is difficult to estimate the selective pressure on genes in natural environments, because the environments in nature are so diverse that the selective pressure is modulated by multiple environmental factors in a complicated manner.

Experimental evolution studies utilize model organisms evolved in defined environments in the laboratory, and therefore they address environmental adaptation more directly. Indeed, previous experimental evolution studies observed genomic alterations under environmental selection and evaluated the effectiveness of multiple genes on fitness [5], [6], [7], [8]. Those molecular studies generally utilized unicellular organisms, such as bacteria and yeast, because of their short generation times and relatively small genomes. Experimental evolution studies using multi-cellular sexual organisms have generally been limited to analyses of trait evolution; for example increased abdominal bristle number in *Drosophila* [9]. Recent progress in genome science, as represented by next-generation sequencing (NGS) technology, has changed the situation by enabling us to determine the whole genome sequences of organisms from enormous output data [10]. This technology has recently been applied in some experimental evolution studies. Burke et al. showed genome sweep in *Drosophila* populations selected for accelerated development [11] and Zhou et al. analyzed genome features of hypoxia-tolerant *Drosophila* populations [12]. NGS is now starting to be used to characterize the whole genome sequences of laboratory-evolved organisms.

**Figure 1:**
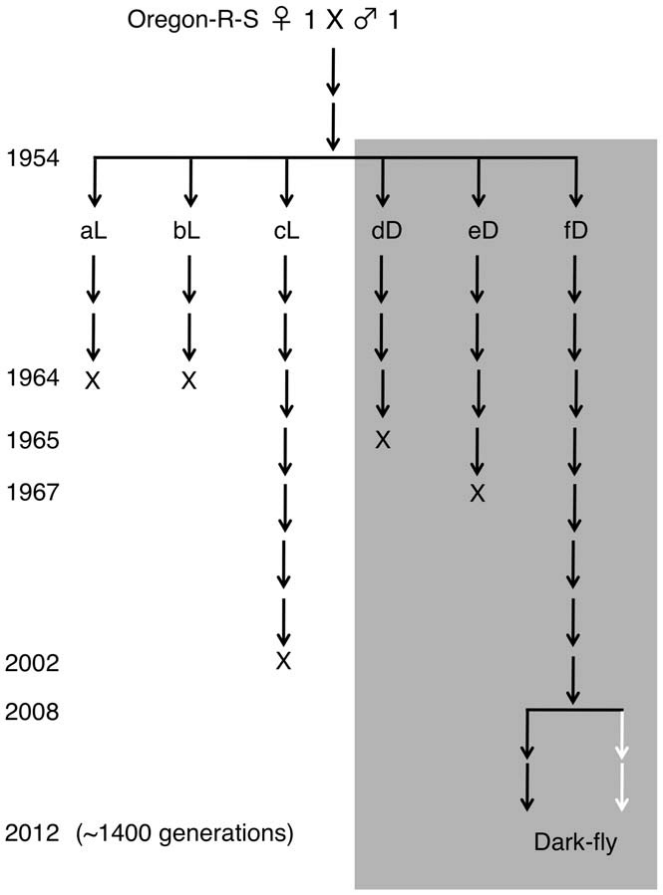
History of Dark-fly. In 1954, a fly population derived from one pair of Oregon-R-S flies was divided into 6 populations. Three of them (aL, bL and cL populations) were reared in normal light-dark cycling conditions and the remaining three populations (dD, eD, and fD populations) were reared in constant dark conditions. Unfortunately, all of the L lines were lost by 2002. The dD and eD lines were lost in 1965 and 1967, and only the fD line has been maintained until now. In 2008, we started to rear the fD line and designated it “Dark-fly”. We have maintained Dark-fly in a minimum medium as done before (black lines), and in a standard cornmeal medium (white lines) in parallel. The population size of Dark-fly has not been controlled but has usually been about 100 flies each in several culture vials.

We utilized NGS technology to study an unusual line of *Drosophila*. On November 11, 1954, the late Dr. Syuichi Mori (Kyoto University) started an experiment of maintaining a *Drosophila melanogaster* strain, Oregon-R-S, in constant dark conditions (Fig. 1) [13]. Through 2012, this fly line, designated Dark-fly Oregon-R-S (hereafter referred to simply as “Dark-fly”) has been reared in darkness for 57 years (1400 generations). Previous studies revealed that Dark-fly showed strong phototactic ability compared to the control sister lines that had been maintained in normal light conditions [14], [15]. It is known that flies reared in the dark become sensitive to light via physiological changes [16]. Interestingly, the phototactic ability of Dark-fly remains high even after rearing in the light for 100 generations [17], indicating that Dark-fly seems to have lost the physiological plasticity of this trait, presumably due to genomic alterations. It was also shown that the head bristles of Dark-fly are longer than those of the wild-type strain [18] and Dark-fly maintains circadian rhythms as well as the control line does [19]. Since Dark-fly possesses eyes and pigmented cuticles and does not show apparent morphological traits related to the adaptation, it is unclear if Dark-fly is really adapted for living in the dark. Unfortunately, the control sister lines were lost during the rearing history, and only one of three replica lines reared in the dark (fD line) has survived until now (Fig. 1). Therefore, it is impossible to compare Dark-fly directly with the control sisters. Nevertheless, Dark-fly is a unique organism reared long-term in a dark environment, and accordingly can be utilized for analyzing traits and genes involved in environmental adaptation. Furthermore, Dark-fly has been reared with a minimal medium, called Pearl's medium [14]. There is a considerable possibility that poor nutrient conditions influence the selective pressure in dark environments. Thus, Dark-fly might be useful for analyzing interactive effects of environmental factors on selection, which probably occur in nature.

Here, we found that Dark-fly produced more offspring in dark than in light conditions, suggesting that Dark-fly possesses some traits advantageous in darkness. To examine genomic alterations involved in environmental adaptation, we performed whole genome sequencing for Dark-fly using NGS technology and found unique features of its genome.

## Results

### Dark-fly produces more offspring in dark than in light conditions

**Figure 2:**
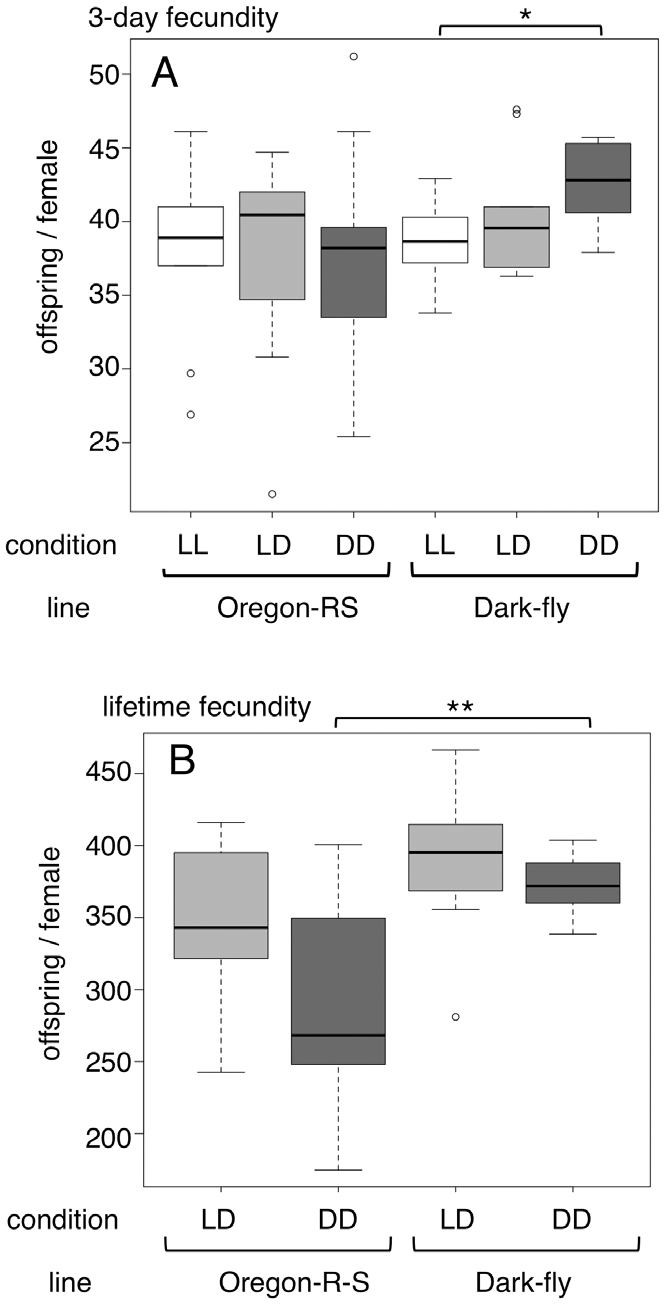
Fecundity of Dark-fly and Oregon-R-S. (A) Three-day fecundity (offspring/female) of Dark-fly and Oregon-R-S in LL, LD and DD conditions are shown by box plots. Boxes and median lines represent inter-quartile range and median values of data, and vertical lines represent minimum and maximum values of data within 1.5-fold of the inter-quartile range. Circles indicate values of outliers. * indicates FDR-adjusted p-value<0.05, Welch t-test. n = 10 (total 100 females). (B) Lifetime fecundity (offspring/female) of Dark-fly and Oregon-R-S in LD and DD conditions are shown by box plots in a similar manner to (A). ** indicates p-value<0.01, Welch t-test. n = 10 (total 100 females).

We first asked whether Dark-fly exhibits successful reproduction in dark conditions, as a feature of environmental adaptation. Adult flies were placed in a light-dark cycling (12-hour ∶ 12-hour; LD), constant light (LL) or constant dark (DD) condition for 3 days and the offspring were counted. We used the Oregon-R-S strain, which was obtained from a stock center, as a control line, because Dark-fly originated from that strain [14]. Oregon-R-S produced approximately 40 offspring/female during 3 days irrespective of whether the flies were tested in the LL, LD, or DD condition (Fig. 2A). In contrast, Dark-fly produced significantly more offspring in the DD condition than in the LL condition (42.6±2.8 in DD versus 38.6±2.6 in LL; Welch t-test, FDR-adjusted p-value = 0.033, n = 10 (total 100 females)). A tendency toward relatively high fecundity in the DD condition was also observed when compared with the LD condition, although the difference was not statistically significant (40.3±4.1 in LD; Welch t-test, FDR-adjusted p-value = 0.195, n = 10 (total 100 females)). These results suggest that Dark-fly produces many offspring in dark conditions over a period of 3 days, but Oregon-R-S does not show such an advantage in the dark.

We next examined the fecundity over a fly's lifetime. Dark-fly produced a similar number of offspring over its lifetime in LD and DD conditions (Fig. 2B). This suggests that the reproductive ability of Dark-fly *per se* is not altered in the dark, but rather Dark-fly produces more offspring early during the mating period (during the first 3 days) in the dark. Oregon-R-S as well as Dark-fly produced approximately 300 offspring/female over its lifetime. It seems that Oregon-R-S decreased the number of offspring produced in the DD compared to the LD condition, but Dark-fly maintained it. Consequently, Dark-fly produced significantly more offspring than Oregon-R-S in the DD condition (373±20 for Dark-fly versus 293±73 for the Oregon-R-S; Welch t-test, p-value = 0.006, n = 10 (100 females)).

**Figure 3:**
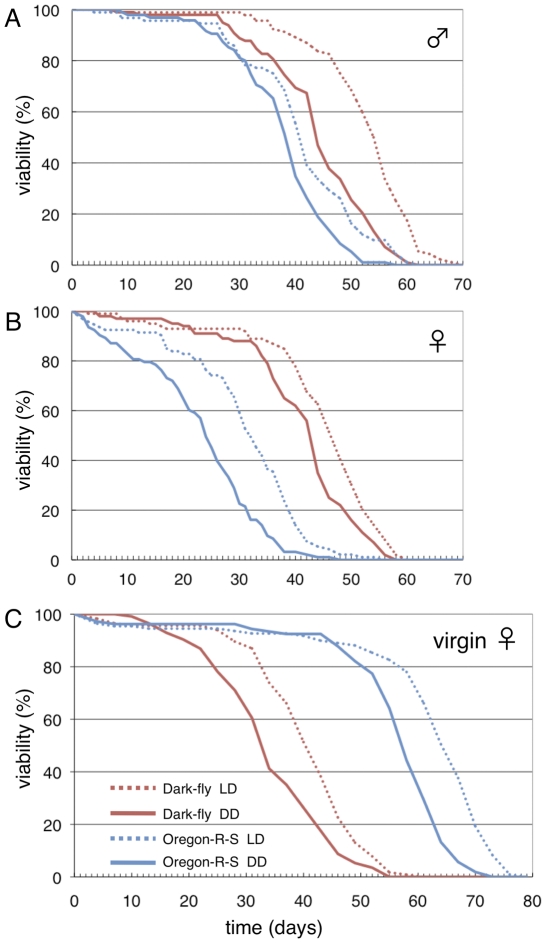
Survival curves of Dark-fly and Oregon-R-S. The viability of male flies (A) and female flies (B) reared together is plotted versus time (days). Dark-fly (red lines) and Oregon-R-S (blue lines) were reared under LD (dotted lines) and DD (solid lines) conditions. The viability of virgin females (C) was also measured in a similar manner. n = 92–100 flies. Oregon-R-S virgin females showed longer longevity than the mated ones, whereas Dark-fly virgin females showed shorter longevity than the mated ones.

The decreased fecundity of Oregon-R-S in the dark appears to be partly due to decreased adult viability. When males and females were reared together, Oregon-R-S and Dark-fly males showed similar viability (Fig. 3A) but Dark-fly females survived longer than Oregon-R-S females in either the LD or DD condition (Fig. 3B). Females of both lines survived longer in the LD condition compared to the DD condition. However, remarkably, Oregon-R-S females gradually died in the DD condition (Fig. 3B, solid blue line), but Dark-fly females did not show such gradual death (Fig. 3B, solid red line). Consequently, the 50% survival period in the DD condition was 43 days for Dark-fly and 24 days for Oregon-R-S. It is unlikely that Dark-fly possesses extraordinary longevity, because Dark-fly virgin females showed shorter longevity than Oregon-R-S virgin females (Fig. 3C). Even more surprisingly, Dark-fly virgin females showed shorter longevity than the mated ones (Fig. 3B, 3C, red lines). It is generally considered that reproduction is a cost for longevity [20], as seen in Oregon-R-S (Fig. 3B, 3C, blue lines). Dark-fly females might not have the cost of reproduction. Thus, Dark-fly females produce offspring earlier and yet maintain longevity in dark conditions. These traits would contribute to the reproductive success in darkness.

### Whole genome sequencing for Dark-fly

To understand the molecular nature of Dark-fly's traits, we extracted genomic DNA from 20 adult males each of Dark-fly and Oregon-R-S, and performed whole genome sequencing using an Illumina Genome Analyzer II. Approximately 67 million and 87 million reads were obtained for Dark-fly and Oregon-R-S, respectively, and 96 and 90% of reads were successfully aligned to the *Drosophila* reference genome (Table 1). Since the read sequence for Dark-fly covered the genome with mean depth of 14, our data were suitable for analyzing the features of the genome comprehensively.

**Table 1:** Summary of genome sequencing.

fly line	read length	read number	mapped read number	mapped read %	total read bases	mean depth
Dark-fly	36	66,855,594	64,422,374	96.4	2,319,205,464	13.7
Oregon-R-S	36, 39, 48	87,101,330	78,109,114	89.7	3,307,906,716	19.6

The results of genome sequencing using an Illumina Genome Analyzer II are summarized. Flybase Dmel 5.22 genome (168,736,537 bases) was used as a reference genome.

After filtering the quality of each sequence, single nucleotide polymorphisms (SNPs) were identified at 415,626 sites for Dark-fly and 415,668 sites for Oregon-R-S, compared with the reference genome sequence (Table 2). Since we judged SNPs by the criterion that the altered nucleotide was found at more than 90% frequency of total reads, these SNPs are likely fixed in the populations. 198,286 SNPs (47.7% for Dark-fly) were shared between the two lines, and 217,340 SNPs were specifically identified in Dark-fly. Although Dark-fly was derived from the Oregon-R-S strain, the genome sequences of the present Dark-fly and the present Oregon-R-S were thus found to be somewhat divergent. This might be explained by several possibilities: for example, the Oregon-R-S strains might have originally been divergent between laboratories (see Discussion). We noted that the “common” and “specific” Dark-fly SNPs were not distributed evenly on the chromosomes, but rather were present in some clusters in mosaic patterns (Fig. S1). This suggests that large-scale genomic alterations, such as inversions and translocations of chromosomal fragments, might have occurred in the Dark-fly genome. We also examined the mitochondrial genome, which is maternally inherited and is not subject to recombination. Twelve of 16 SNPs (75%) found in Dark-fly corresponded to those of Oregon-R-S (12 of 19), suggesting that the maternal origins of the two lines were related. To understand how close the Dark-fly genome is to the Oregon-R-S genome, we compared them with genomes of a group of other lines (the DGRP lines) [21], which are inbred lines generated from a natural population (see Materials and Methods). Phylogenetic tree analysis revealed that the DGRP lines are highly diverse, whereas Dark-fly and Oregon-R-S are relatively close (Fig. S2), suggesting that although the present Dark-fly has many SNPs compared to the present Oregon-R-S, these two lines are closely related.

**Table 2:** SNP, InDel and CNV analyses.

fly line	Dark-fly	Oregon-R-S
total fixed SNPs	415,626	415,668
SNP frequency (bases/SNP)	406	406
line-specific SNPs	217,340	217,382
total SNPs		
total SNP-effects	1,435,028	1,424,012
intergenic	826,111	824,781
UTR and intron	486,090	499,604
synonymous coding (sSNP)	96,674	78,152
non-synonymous coding (nsSNP)	25,514	20,840
others	639	635
line-specific SNPs		
nsSNPs without redundancy	9,695	6,521
genes carrying nsSNPs	4,323	3,039
genes carrying nonsense mutations	28	23
total fixed InDels	5,322	5,461
InDel frequency (bases/InDel)	31,705	30,898
line-specific InDels	4,660	4,799
total InDels		
total InDel-effects	16,726	17,507
intergenic	8,790	9,767
UTR and intron	7,790	7,674
coding region (cInDel)	144	66
others	2	0
line-specific InDels		
cInDels without redundancy	52	27
genes carrying cInDels	50	27
genes showing increased CNVs	122	ND
genes showing decreased CNVs	133	ND

These data represent a summary of our analyses of SNPs, InDels and CNVs for the Dark-fly and Oregon-R-S genomes. ND means not determined.

### Non-synonymous SNPs and coding InDels were concentrated in some gene families

Since Dark-fly displays some traits advantageous for living in the dark, it should carry some genomic alterations related to these traits. Even if so, most of the SNPs we found would be expected to be functionally neutral and only a small fraction of the SNPs should contribute to the traits. To evaluate the Dark-fly SNPs, we categorized each SNP by its position relative to gene structures, such as intergenic regions and gene coding regions. Since one SNP often affects several isoforms of a gene or several overlapping genes simultaneously, the 415,626 SNPs of Dark-fly were classified to 1,435,028 SNP-effects (Table 2). It is not easy to evaluate SNPs in intergenic regions, and accordingly we focus on the coding SNPs hereafter. 6.7% of the SNP-effects were synonymous SNPs (sSNPs: i.e., they do not alter amino acid sequences of gene products), and 1.8% were non-synonymous SNPs (nsSNPs: i.e., they change the amino acid sequence) (Table 2). We collected the Dark-fly-specific nsSNPs without redundancy between isoforms and identified 4,323 genes carrying nsSNPs. We performed similar processes for the Oregon-R-S genome and identified 3,039 such genes.

An InDel is an insertion or deletion of a few nucleotides and can be detected by analyzing the NGS data. We identified 5,322 and 5,461 InDels for Dark-fly and Oregon-R-S, respectively, and 662 of these InDels (12.4% for Dark-fly) were shared between them (Table 2). We classified each InDel by its position relative to gene structures, by a process similar to that performed for SNP analysis. InDels in gene coding regions (cInDels) would result in codon-deletion, codon-insertion, or frame-shift of gene products, so that the effects of cInDels would be severe, like those of nsSNPs. We identified 50 and 27 cInDels specifically found in Dark-fly and Oregon-R-S, respectively (Table 2).

We then asked whether the nsSNP or cInDel-carrying genes are concentrated in any gene families in the Dark-fly genome. Using the web-based tool DAVID [22], we identified 20 Gene Ontology (GO) families (by molecular function category) that contained nsSNPs or cInDels at higher probability than the average for all genes throughout the genome (p-value<0.05, Table S1). Among them, 4 GO families, including families associated with metal ion binding (GO:0046872) and UDP-glycosyltransferase activity (GO:0008194), were shared between Dark-fly and Oregon-R-S (* in Tables S1 and S2), suggesting that these genes might have been commonly subject to mutations. The remaining 16 GO families were found specifically for Dark-fly (Table S1). These include families associated with carboxylesterase activity (GO:0004091) and guanyl-nucleotide exchange factor activity (GO:0005085). Thus, these gene families have accumulated nsSNPs and cInDels in the Dark-fly genome.

### Nonsense mutations were identified in the Dark-fly genome

Among nsSNPs, a nonsense mutation produces a stop codon in the amino acid sequence of a gene product, and may severely affect the protein's function. We identified 28 nonsense mutations in the Dark-fly genome (Table S3). Among them, 10 mutations (for example, in the Hn and HisCl1 genes) were located in a subset of a gene's isoforms, so that the nonsense mutation might be complemented by redundant function(s) of other isoform(s). The remaining 18 mutations were located at sites shared by all of the gene's isoforms or at sites of the gene encoding a unique transcript, so that functional consequences of these mutations would be inevitable. These genes included an olfactory receptor (Or65c) and a light receptor (Rh7) genes. Indeed, the Dark-fly nonsense mutations were preferentially concentrated to one GO family associated with sensory perception (BP_5 category: GO:0007600, data not shown). We also detected a similar number of nonsense mutations (23 mutations) in the Oregon-R-S genome (Table S4), but those were not concentrated to any GO families.

### Identification of runs of homozygosity regions

**Figure 4:**
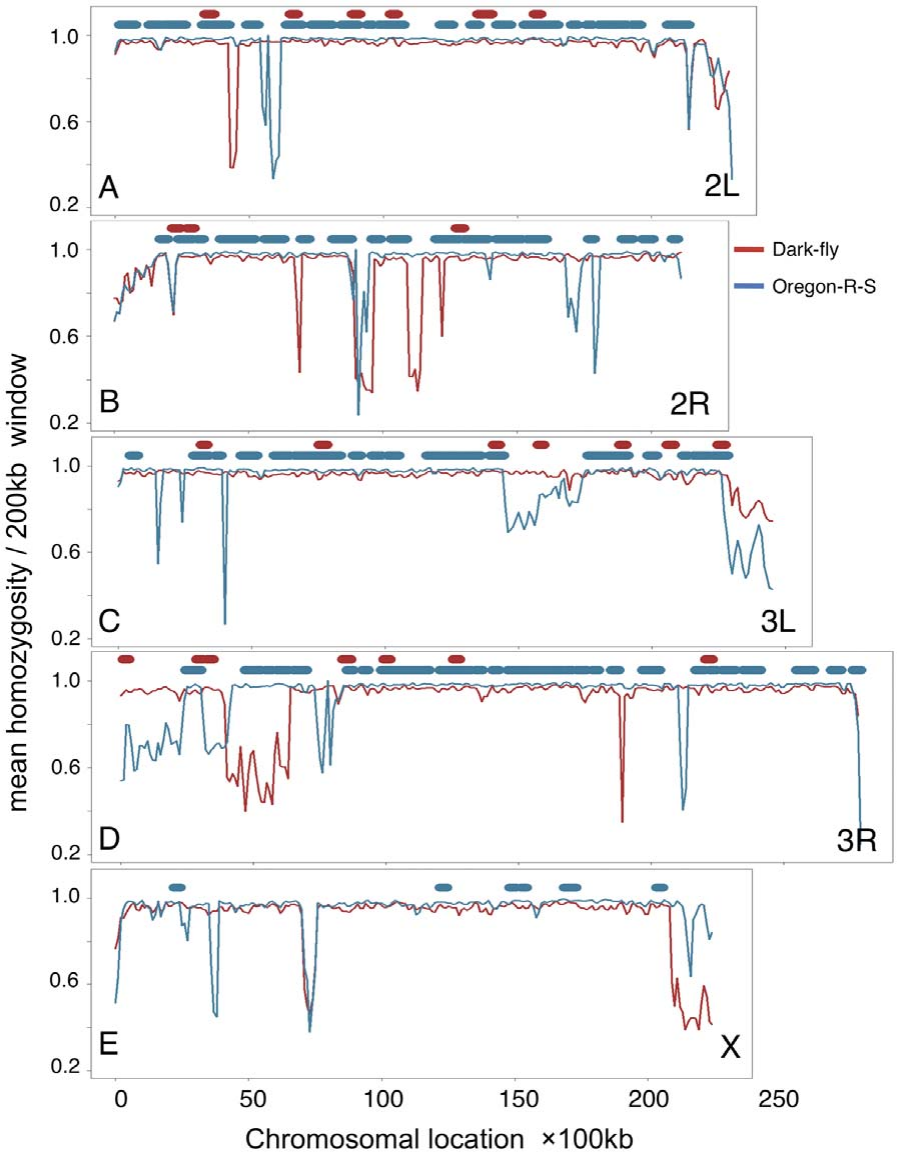
Homozygosity and ROH regions. Mean homozygosity of SNPs in a sliding window (200-kb window at 100-kb steps) was plotted versus the location on 2L (A), 2R (B), 3L (C), 3R (D) and X (E) chromosomes. The Oregon-R-S genome (blue lines) displayed higher homozygosity than the Dark-fly genome (red lines) in most of the regions. Thick horizontal bars represent ROH regions identified by PLINK software for Oregon-R-S (blue bars) and Dark-fly (red bars) and are plotted above the graph without homozygosity values.

Runs of homozygosity (ROH) regions are homozygosity-extended genomic regions (more than a few hundreds kb) containing consecutive homozygous SNPs and are thought to be regions currently selected in a population's genome [23]. This criterion has successfully identified disease-related recessive mutations and positively selected genes in human populations [24], [25]. We expected that the Dark-fly genome might contain homozygosity-extended regions as signatures of historical selections during the 1400 generations. Since our NGS data were obtained from the genomic DNA of 20 flies and cover the genome with 14-fold depth, we considered that our data would be useful to detect ROH regions in the population genome. We listed homozygous SNPs (homo SNPs; frequency greater than 90%) and heterozygous SNPs (hetero SNPs; frequency greater than 40% and less than 90%) from the Dark-fly genome data and identified 449,684 homo SNPs and 28,132 hetero SNPs (Table 3). The overall fraction of homo SNPs was 94.1%, indicating that the Dark-fly genome contains only a small number of hetero SNPs compared to homo SNPs. Using PLINK software [26], we searched homozygosity-extended regions (400 kb sliding window at 200 kb steps) on major chromosomes (2L, 2R, 3L, 3R and X) and identified 24 ROH regions (Fig. 4, Table S5). The total length of ROH regions covered approximately 6 Mb (5% of the genome length of major chromosomes), suggesting that homo SNPs are abundant but ROHs are rare in the Dark-fly genome. We performed a similar process for Oregon-R-S and identified 128 ROH regions that covered approximately 44 Mb (37% of the genome length of major chromosomes) (Fig. 4, Table S6). Thus, although the percentages of homo SNPs were similar between Dark-fly and Oregon-R-S (94.1% versus 93.3%), the ROH number and coverage were clearly different between them (Table 3). This indicates that homo and hetero SNPs are highly clustered in the Oregon-R-S genome but are distributed more evenly in the Dark-fly genome, resulting in the presence of many ROHs in Oregon-R-S and few ROHs in Dark-fly. These genome features might reflect the differences of population history (see Discussion).

**Table 3:** Identification of ROH regions.

fly line	Dark-fly	Oregon-R-S
homo and hetero SNPs (0.4 = <freq)	477,816	486,013
homo SNPs (0.9 = <freq)	449,684	453,646
hetero SNPs (0.4 = <freq<0.9)	28,132	32,367
homo SNP fraction in total (%)	94.1	93.3
number of ROH regions	24	128
total length of ROHs (kb)	5,934	43,868
fraction of ROHs in genome (%)	4.99	36.85
average length of ROH (mean ± SD, kb)	230±70	342±155
average SNP number in ROH (mean ± SD)	981±449	1621±989
average homo SNP fraction in ROH(mean ± SD, %)	97.5±0.9	98.2±0.5
number of ROH regions with significantly high homozygosity	21	ND
genes carrying nsSNPs and cInDels in ROH regions	241	ND

These data represent a summary of our analyses of ROH regions. Homo and hetero SNPs were identified using Samtools and Vcftools functions. The number of homo SNPs was slightly different from that of the fixed SNPs identified using VarScan functions (Table 2), due to the difference of data filtering. ROH regions were identified using PLINK software (Tables S5 and S6). The Dark-fly ROH regions showing significantly high homozygosity were determined by statistical analyses (Tables S7 and S8). Genes carrying nsSNPs and cInDels in 21 ROH regions were counted. ND means not determined.

We also measured mean homozygosity (mean frequency of each SNP) in the Dark-fly and Oregon-R-S genomes (Table S7). The mean homozygosity of the Oregon-R-S genome was slightly higher than that of the Dark-fly genome (0.944 in Oregon-R-S versus 0.941 in Dark-fly). Sliding window analysis revealed that in both lines, high homozygosity was expanded widely throughout the genome and only a small number of regions showed low homozygosity (Fig. 4). This seems to be a genome feature of inbred organisms. In most genomic regions, the Oregon-R-S genome displayed higher homozygosity than the Dark-fly genome, consistent with the difference of ROH number and coverage (see Fig. 4 blue and red lines). To evaluate the Dark-fly ROH regions statistically, we compared the mean homozygosity of each ROH region with the average homozygosity of the whole genome (Table S8). Three of the 24 ROH regions (ROH ID#8, 12 and 18) failed to be significantly different from the average (Table S8; Welch t-test, p-value<0.01), probably due to the presence of some SNPs with low homozygosity. Statistical analysis of the enrichment of homo SNPs in each ROH region using Fisher's exact test also yielded the same result (Table S8). Taking these data together, we identified 21 ROH regions showing significantly high homozygosity in the Dark-fly genome (Table 4). We suggest that these ROH regions might be genome signatures selected in the Dark-fly population.

**Table 4:** Genes carrying nsSNPs and cInDels in the Dark-fly ROH regions.

ROH ID#	Chr	positionstart	positionend	lengthbases	genes carrying nsSNPs or cInDels
ROH1	2L	3353705	3669168	315463	CG8838, CG34394, Ptpa, CG34175, CG31952, CG3238, CG31776, Sr-CIV, Spindly
ROH2	2L	6535198	6782752	247554	CG9596, CG11319, CG11320, CG34345, Oatp26F, Tango1, CG31633, CG11070, CG13771, Nhe3, CG11327, GRHR, CG11188, homer, TTLL3A, CG31910, CG11221, CG11322, CG11321, CG17378
ROH3	2L	8847085	9109796	262711	CG32986, CG34398, CG9510, CG31886, CG32985, CG32984, CG18088, CG9541, CG9555, CG17906, CG18661, CG9568, CG9582, Toll-4
ROH4	2L	10278630	10524864	246234	CG34043, CG5604, CG13138, CG5384, CG4972, GATAd, CG34367, CG5367, Cand1, pim, CG5056, rho-5, CG33303, gny, CG5168, CG5188, CG6232, CG5322, CG6206, RluA-1, RluA-2, CG7456, CG13144, Myo31DF, CG7384, Fatp
ROH5	2L	13521459	13806482	285023	CG33641, CG33644, CG33645, CG16853, CG18507, CG7311, CG31814, CG9014, CR31845, CG31731, sec71
ROH6	2L	13806743	14034237	227494	CG16865, Sos, b, tam, Orc5, mRpS23, CG33307, CG33306, CG8997, cenG1A, Ance-2, CG16886, CG16884, nimB1, nimB3, nimB5, He, nimC1, rk, bgm, CG18095
ROH7	2L	15628469	15854613	226144	CG7631, CG18480, CG4587, CycE, Ku80, CG18109, CG18518
ROH9	2R	2722221	2975600	253379	CG15236, Spn42Db, Spn42De, CG3358, CheB42b, CheB42c, ppk25, mim, Cyp6u1, CG30157, vimar, Tsp42Ee, Tsp42Eh, Tsp42Ei, CG12831
ROH10	2R	12738094	13006423	268329	Fen1, CG8910, Pkc53E, CG15614, mute, CG6665, ste24b, CG6796, CG8963, Ark, RhoGEF2, CG9640, CG9642, CG9646, CG8950, CG6967, CG30460, CG30456, CG15611
ROH11	3L	3118085	3327625	209540	CG14963, CG32284, CG32277, CG12034, CG11505, CG12009
ROH13	3L	14059399	14275678	216279	pex1, CG8100, Fbp1, Sox21b, nuf, CG34244
ROH14	3L	15737620	15945049	207429	CG13445, CG12713, CG32150, CG12486, pHCl, sff, Pka-C3
ROH15	3L	18793182	19024297	231115	CG14073, CR32027, CG14074, dysb, CG11637, Ir75d, CG14077, CG3819, CG14075, CG11619, CG18135, CG3808, CG18136, nkd
ROH16	3L	20560665	20819130	258465	CG13251, CG34260, CG13252, CG4074, Pitslre, Spc105R
ROH17	3L	22471441	22725139	253698	CG14459, CG14453, CG11370, CG6838, CG32454
ROH19	3R	2862778	3085343	222565	CG1988, CG1105, CG1965, CG1943, CG1091, CG31248, MAGE, lap, CG14605, CG1227
ROH20	3R	3257401	3475620	218219	CG14598, alpha-Est10, alpha-Est9, alpha-Est8, alpha-Est7, alpha-Est6, alpha-Est5, alpha-Est3, alpha-Est2, CG34127
ROH21	3R	8358059	8659641	301582	Octbeta2R, CG11608, Cyp313a4, CG14391, mus308, Men, CG5724, CG5999
ROH22	3R	9912039	10141059	229020	CCHa1, Or88a, Kif19A, 140up, CG14356, CG42500, CG31533, CG31327, DopR, CG9649, CG9631, Aats-met, trx, CG3259, su(Hw), CG31321
ROH23	3R	12540659	12771162	230503	Ubx, Glut3, Abd-B
ROH24	3R	22056540	22307403	250863	CG14239, Hex-t1, CG5455, CG6490

The chromosomal position and length of the Dark-fly ROH regions showing significantly high homozygosity are listed. Genes carrying nsSNPs and InDels in each ROH region are shown. Details regarding nsSNPs and cInDels are presented in File S1.

### nsSNPs and cInDels in ROH regions

We further characterized the Dark-fly ROH regions and identified 241 genes containing nsSNPs and/or cInDels (Table 4). GO analysis for the 241 genes listed 3 families (Table S9). One of them is associated with carboxylesterase activity (GO:0004091), and two of them are related families associated with small GTPase regulator activity (GO:0005083) and guanyl-nucleotide exchange factor activity (GO:0005085). Interestingly, both families of carboxylesterase and guanyl-nucleotide exchange factor were also listed by the aforementioned GO analysis of total nsSNPs and cInDels (Table S1). Carboxylesterase genes are located as a cluster at the ROH ID#20 region on chromosome 3R (Table 4). Carboxylesterase is a family of the enzymes hydrolyzing esters, and the alpha-esterase class listed here is involved in xenobiotic matabolism [27]. Guanyl-nucleotide exchange factors (GEFs) are regulators of small GTPases involved in various biological processes, such as neural development and activity [28]. These and other genes that carry nsSNPs and cInDels in the ROH regions are potential candidate genes related to the selected traits of Dark-fly (Table 4, File S1).

### CG4594 gene is deleted in the Dark-fly genome

**Figure 5:**
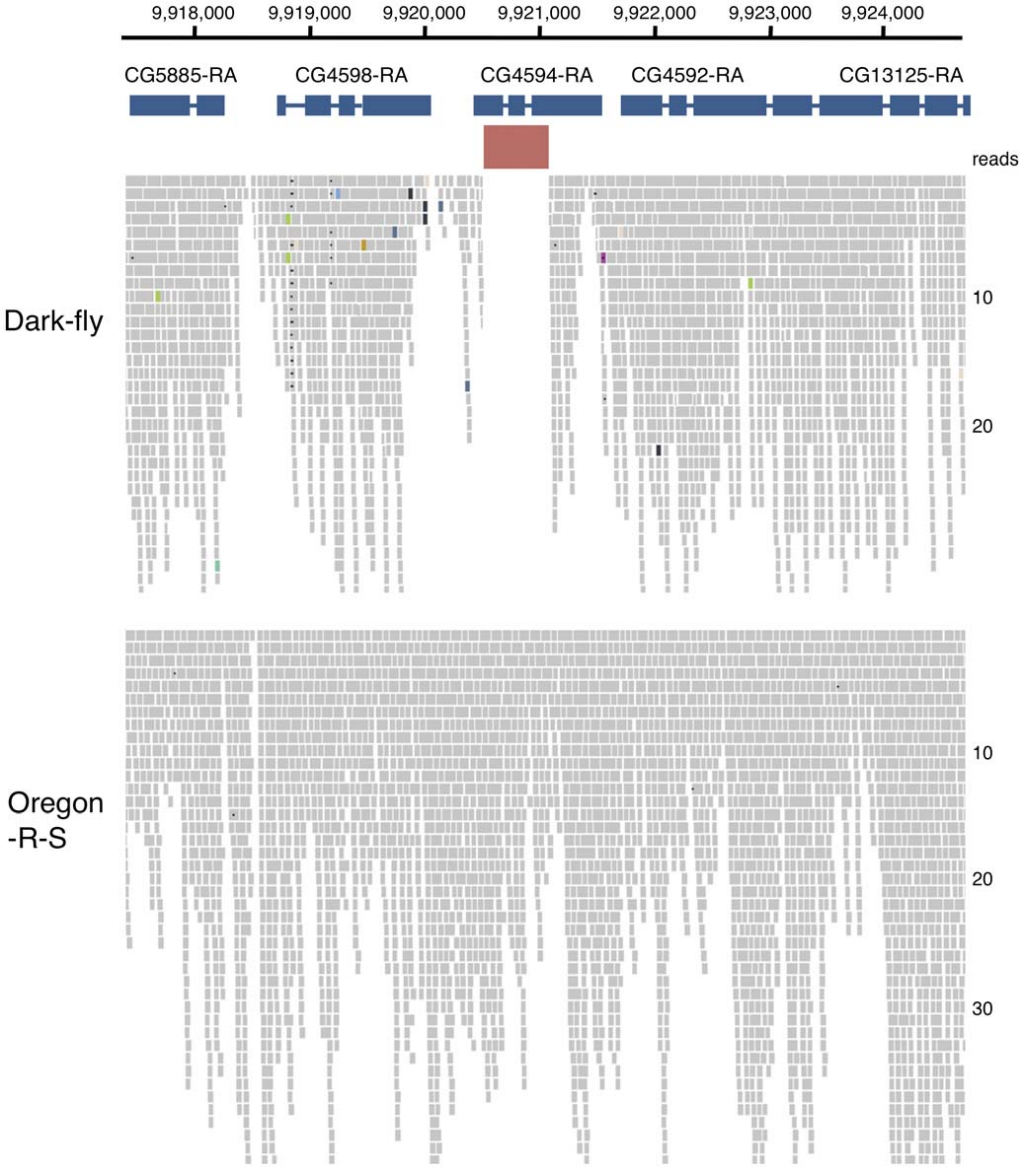
Alignment of read sequences around CG4594 gene. A view of Integrated Genomics Viewer around the CG4594 gene. The numerous small gray bars represent reads of genome sequencing. A region of about 500 bases in the CG4594 gene (red thick bar) was not covered by any read sequences of the Dark-fly genome (upper), but was fully covered by the sequences of the Oregon-R-S genome (lower). Numbers on a horizontal line indicate nucleotide position on chromosome 2L. Numbers on vertical alignment indicate read depth.

Structural variations are generated by recombination and transposition of genome fragments, and together with SNPs and InDels, are important types of genomic alterations. Since short-read sequencing by NGS technology is not suitable for analyzing large-scale structural variations, we instead performed microarray analysis of genomic DNA. We used a *Drosophila* array platform spotted with approximately 18,000 probes that corresponded to coding regions for almost all genes. We compared the Dark-fly genome with the control genome to detect increased and decreased signals as copy number variations (CNVs). After strictly filtering the quality of the data, we analyzed 4,000 probes and identified 122 genes with increased CNVs (iCNVs) and 133 genes with decreased CNVs (dCNVs) (cut-off p-value<0.01) (Table 2, File S2). It is possible that the genome fragments including these genes are duplicated or deleted in Dark-fly. Alternatively, SNPs and InDels might be highly accumulated in these genes, and consequently the ratio of array signals would be increased or decreased. We examined the sequence alignments of NGS data for each gene detected as a dCNV, and thereby found a deletion of at least one gene. As shown in Figure 5, a region of about 500 bases in the CG4594 gene was not covered by any read sequences of the Dark-fly genome. This was not due to problems of the sequencing procedure or alignment process, because the region was fully covered by sequences of the Oregon-R-S genome. These two independent types of evidence (CNV data and NGS data) strongly suggest that the coding region of CG4594 is deleted in the Dark-fly genome. The CG4594 gene encodes a putative dodecenoyl-CoA delta-isomerase. Although the role of this gene is unknown, homologous mammalian enzymes are involved in fatty acid metabolism inside the mitochondria [29].

## Discussion

### Reproductive success in dark conditions

Reproductive success is one of the adaptive traits under natural and laboratory selection. Dark-fly produced more offspring in the dark than in the light for the first 3 days. This early reproduction of Dark-fly would be advantageous in the laboratory routine of fly maintenance. We observed that Dark-fly females do not show the gradual death that occurs in Oregon-R-S females in the dark, and as a result, Dark-fly females retain fecundity for a longer time in the dark. This trait would also contribute to reproductive success.

The early reproduction could be achieved via various traits of the fly, for example, egg-laying ability and mating behavior. Indeed, we observed abnormal mating behaviors of Dark-fly. Dark-fly males and females copulated more quickly than the Oregon-R-S pairs (K. Okamoto and N.F., unpublished data), suggesting that mating behaviors might be stimulated in the Dark-fly pairs: males might easily become active for courtship and females might easily accept males. Mating behavior is controlled by multiple sensory inputs, such as smell and taste [30], [31]. One hypothesis is that Dark-fly might be sensitive to sensory signals, for example, sexual pheromones. Since the quick copulation of Dark-fly was observed in light conditions as well as in dark conditions (K. Okamoto and N.F., unpublished data), the quick copulation alone would not account for the early reproduction in the dark. However, we speculate that stimulated sexual behavior contributes to the early reproduction via re-courtship after failure and also via repeated mating.

Oregon-R-S females gradually died in dark conditions, while Dark-fly females did not show such gradual death. This phenomenon is probably a complex consequence not easily explained, but it might be related to the fact that Dark-fly females retain longevity after mating. Reproduction is generally a cost for longevity [20], and in accord with this, Oregon-R-S virgin females showed much longer longevity than the mated ones. The cost of mating for females is thought to be an advantage for males because it prevents the production of offspring of other males. During copulation, a male transfers seminal fluid containing ACPS protein to a female, and ACPS protein influences the metabolism and physiology of females [32]. It has also been proposed that some volatiles emanated from males cause deleterious effects on females without mating [33]. We speculate that Dark-fly females might be resistant to such deleterious compounds, and that Oregon-R-S females might be sensitive to them, especially in the dark. Alternatively, these phenomena might be due to the traits of males; for example, seminal fluids of Dark-fly males might not be deleterious to females.

### Genome history of Dark-fly

We determined the whole genome sequence for Dark-fly and identified approximately 220,000 SNPs and 4,700 InDels compared with the genome of Oregon-R-S strain. Although Dark-fly was derived from the Oregon-R-S strain 57 years ago, the genome sequences of the present Dark-fly and the present Oregon-R-S were somewhat divergent. Previous studies evaluated the spontaneous nucleotide mutation rate in *Drosophila* and estimated it to be 1/10^9^ to 1/10^8^ per nucleotide per generation [34], [35], which is a value that is approximately conserved among diverse organisms [36]. Given that most newly arisen mutations have been fixed in a relatively small population (about 100 flies) of Dark-fly, we estimated that 400–4000 mutations would arise during 1400 generations by a simple calculation: mutation rate (1/10^9^ to 1/10^8^)×genome size (1.5×10^8^ bases×2)×generations (1400 generations). Therefore, the number of SNPs found between Dark-fly and Oregon-R-S would be 55 to 550 times greater than the predicted number, if two lines had been derived from exactly the same ancestor. This discrepancy might be explained by several possibilities. The Oregon-R-S strains might have originally been diverse in the stocks in different laboratories. Another possibility is that the mutation rate in one of the strains was accelerated, for example via mutation in a DNA polymerase enzyme [5]. Alternatively, unexpected contamination might have occurred during the history of the strains. It is impossible to distinguish among these possibilities at present, because we have neither the original fly from 57 years ago nor sister lines maintained in parallel with Dark-fly (Fig. 1). To better understand how close or dissimilar the Dark-fly genome is to the Oregon-R-S genome, we compared them with genomes of other inbred lines (the DGRP lines) [21]. Phylogenetic analysis revealed that Dark-fly and Oregon-R-S are much closer compared to various DGRP lines derived from a natural population. We therefore suggest that although Dark-fly has many SNPs when compared to Oregon-R-S, the two lines are near relations.

Analyses of ROH regions unexpectedly revealed that although the Dark-fly and Oregon-R-S genomes contain similar numbers of homozygous (fixed) and heterozygous (floating) SNPs, they contain different numbers of homozygosity-extended regions. That is, whereas fixed SNPs and floating SNPs are clustered with each other in the Oregon-R-S genome, they are distributed more evenly in the Dark-fly genome. These genome features might reflect differences of the population histories. For example, inbreeding (isogenization) might have occurred frequently for Oregon-R-S during its history, and consequently many SNPs might have become fixed as clusters in the population genome. In contrast, Dark-fly has been maintained mostly as a constant population size (about 100 flies), and many genomic regions might still be under genetic drift. If this is true, it would strongly support the notion that the Dark-fly ROH regions are rare genome regions selected during the current history (57 years).

### Candidate genes possibly involved in Dark-fly's traits

Dark-fly possesses some traits advantageous in darkness and should carry some genomic alterations responsible for these traits. To search for such mutations, we characterized SNPs, InDels, and CNVs in the Dark-fly genome. We identified 21 ROH regions selected during the Dark-fly history. These regions contain 241 genes carrying nsSNPs and cInDels. These genes include 9 alpha-esterase genes, which are located as a cluster on chromosome 3R [37]. Alpha-esterases are involved in the metabolism of xenobiotics (so-called detoxification) [27]. Although the targets of each alpha-esterase are still unclear, some alpha-esterases function in resistance against pesticides, such as organophosphates [38]. Interestingly, GO analysis of total nsSNPs and cInDels listed another gene family related to detoxification, UDP-glycosyltransferase (UGT) genes [39], as well as the esterase family. The UGT family was listed for both Oregon-R-S and Dark-fly, though the mutation rate in this gene family was higher in Dark-fly (compare count numbers in Tables S1 and S2). Thus, Dark-fly nsSNPs and cInDels are concentrated in two detoxification enzyme families. It is known that alpha-esterase and UGT genes are expressed under circadian regulation in *Drosophila* as well as in other animals [40]. Indeed, flies' resistance against pesticides oscillates daily [41]. Although a previous study showed that locomotor activity of Dark-fly displays normal circadian rhythm [19], the intriguing question of whether detoxification rhythm is changed in Dark-fly has not yet been answered. The biological meaning of detoxification rhythms is still mysterious, but they are expected to promote cost-effective performance during feeding time, when flies are exposed to chemical compounds from the environment. We also speculate that light itself might influence the detoxification process. It is known that bilirubin, a human xenobiotic derived from heme, is metabolized by UGT and that light exposure bypasses the requirement for UGT in this process [42]. Dark-fly might possess specialized metabolism of xenobiotics in light-free conditions. It is also known that some vertebrate detoxification enzymes are preferentially expressed in olfactory epithelium and act on the clearance of odors after perception [43]. Similarly, some *Drosophila* enzymes are expressed in the olfactory organ [44]. We speculate that the detoxification enzymes might be related to olfactory ability in Dark-fly.

The Dark-fly ROH regions also contain 5 guanyl-nucleotide exchange factor (GEF) genes carrying nsSNPs and cInDels. GEFs are regulators of small GTPase involved in various biological processes, such as neural development and activity. For example, Son of sevenless (Sos) is required for development of R7 photoreceptor neurons [45] and is also involved in circadian rhythms of clock neurons [46]. RhoGEF2 organizes the morphology of cells and functions in axonal growth [47]. Recently, Yuan et al. found that the morphology of larval photoreceptor neurons is plastically changed by light and dark conditions [48]. An intriguing issue for future studies is whether Dark-fly retains this neural plasticity.

We identified 28 nonsense mutations in the Dark-fly genome (Table S3). Among them, 18 mutations are considered to alter all of the gene's products, so that the functional consequences of these mutations would be serious. These genes include one encoding an olfactory receptor (Or65c). It has been proposed that olfactory receptor genes evolve rapidly in a non-neutral manner, and often become pseudogenes [49]. According to this notion, mutations of these genes would generate diversity of odor discrimination between species and even between individuals. In the Dark-fly genome, we detected nsSNPs in 36 of 59 olfactory receptor (Or) genes (data not shown), in addition to the nonsense mutation in the Or65c gene. These mutations might be related to odor discrimination of Dark-fly.

Rhodopsin is a light-sensing receptor that belongs to the G protein-coupled receptor family, and the *Drosophila* genome encodes 7 rhodopsins [16], [50]. The Dark-fly genome contains a nonsense mutation in the rhodopsin7 (Rh7) gene but no nsSNPs in other rhodopsin genes (data not shown). Although the *in vivo* functions of Rh7 are still unclear, it is known that the Rh7 protein possesses a unique structure: both its N- and C-terminal regions are longer and its third cytoplasmic loop is shorter than those of other rhodopsins. A nonsense mutation in Dark-fly is located in the C-terminal region (Table S3) and results in the truncation of 21 amino acids from the C-terminus of the wild-type Rh7 protein (483 amino acids long). We suggest that the long C-terminal region plays some roles in the functions of Rh7 because the entire amino acid sequence of the Rh7 protein is highly conserved between the *Drosophila* genus and some other insects (O.N. and N.F., unpublished data).

The independent lines of evidence of our CNV data and our NGS data strongly suggest that the coding region of CG4594 is deleted in the Dark-fly genome. The CG4594 gene encodes a putative dodecenoyl-CoA delta-isomerase. In *Drosophila*, 5 genes (CG4594, CG4592, CG4598, CG5844 and CG13890) encode putative dodecenoyl-CoA delta-isomerases, but their functions have not been characterized so far. It is known that the homologous mammalian enzyme catalyzes a step in the synthesis of acetyl-CoA from fatty acid inside mitochondria and is involved in energy homeostasis [29]. Acetyl-CoA is not only a source of energy but also a compound used in the synthesis of juvenile hormone in *Drosophila* [51]. The deletion of the CG4594 gene in Dark-fly might affect the energy production and/or the hormonal regulation of the fly's physiology.

We identified ROH regions selected in the Dark-fly genome, and found that nsSNPs and cInDels were preferentially accumulated in some gene families in these regions. These are potential candidate genes related to Dark-fly's traits. Some of the genes might contribute to gain of useful traits or loss of useless traits in the dark environment. Alternatively, some genes might contribute to trade-off between useful traits and useless traits, as demonstrated in cavefish: the cavefish Shh gene has pleiotropic roles for gain of a wide jaw and loss of eyes [52]. Further analyses of candidate genes will clarify the effects of these mutations in Dark-fly. Since we evaluated SNPs, InDels and CNVs using limited criteria, we have not excluded the possibility that other (coding and noncoding) mutations not discussed here contribute to the environmental adaptation. Also, since Dark-fly has been reared with a minimal medium, it is possible that Dark-fly might be adapted to poor nutrients as well as to the dark, and the genomic alterations we found might be related to the adaptation to the nutrient state. The whole genome sequencing reported here is a first step toward linking genome, trait and adaptation. As a second step, we are now maintaining large mixed populations of Dark-fly and Oregon-R-S in different conditions and will examine the dark-selected SNPs in the population genome. Another intriguing future issue is whether Dark-fly has an altered profile of gene expression. NGS technology will be useful for these experiments, and will provide us a wide array of approaches for experimental evolution studies.

## Materials and Methods

### Flies

Dark-fly Oregon-R-S (referred to simply as “Dark-fly”) was kindly provided by Dr. Michio Imafuku (Dept. of Zoology, Kyoto University). Since 1954, Dark-fly has been maintained in a constant dark condition with a minimal nutrient medium, Pearl's medium (Fig. 1) [14], [53]. In 2008, we started to rear Dark-fly (then at 1351 generations) in a constant dark condition (DD condition) at 25°C with a standard cornmeal medium (80 g cornmeal, 40 g dry yeast, 32 g wheat germ, 50 g D-glucose, 9.6 g agar, 0.4 g butyl benzoate, 4 ml propionic acid/1 liter water). The flies were exposed to dim red light only while newly emerged flies were being transferred to new culture vials. Before the fecundity and viability assays, Dark-fly was reared under light-dark cycling conditions (LD condition: 12-hour cycles) for 3–20 generations to examine the genetically fixed traits.

We used several wild-type strains as controls. The Oregon-R-S strain provided by Dr. Michio Imafuku was derived from the Kyoto Stock Center and was used for analyses of the whole genome sequence. Another Oregon-R-S strain and the Oregon-R strain (the mother strain of Oregon-R-S) obtained from the Bloomington Stock Center (BL#4269 and 25211 stocks, respectively) were used for the fecundity and viability assays and for the CNV analysis, respectively.

### Fecundity and viability assays

Healthy virgin males and females were collected by brief ice-anesthesia 2 days before the experiment. Ten male and 10 female flies were mixed in a culture vial and were reared in constant light (LL), LD or DD conditions for 3 days (72 hours). Offspring were continuously reared in the indicated conditions and were counted after adult emergence.

To measure the lifetime fecundity, flies were reared in LD or DD conditions and were transferred to new vials every one or two days until all of the adults died. The offspring were reared in the LD condition, and the number of pupae was counted as offspring.

To measure the adult viability, 10 flies each in 10 vials were transferred to new vials every one or two days until all of the adults died. Dead adult flies were counted at the time of every transfer. When the total number of dead adults was smaller than the number of flies at the start, flies that had escaped during experiments (less than 8/100) were ignored for the calculation of viability.

Statistical analyses were performed using t.test (with var.equal = F option), pairwise.t.test (with p.adj = “fdr”, var.equal = F options) and boxplot functions of R software (ver. 2.12.1: http://www.r-project.org/).

### Genome sequencing

Genomic DNA was extracted from 20 adult males by a standard method. Briefly, flies were homogenized in lysis buffer (50 mM Tris-HCl pH 7.5, 350 mM NaCl, 10 mM EDTA, 2% SDS, 7 M urea) and the lysate was extracted with phenol and chloroform, and after RNase treatment, genomic DNA was precipitated with ethanol. Sequencing libraries (paired-end library for Dark-fly and single-end library for Oregon-R-S) were constructed according to manufacturer's protocols. Sequencing was performed using an Illumina Genome Analyzer II, and running 8 lanes for each library. Raw sequence data (36, 39 or 48 bases/read) were obtained as FASTQ files. The data were deposited in DDBJ under accession number DRA000451 (DRR001444–DRR001447).

### SNP and InDel calling

*In silico* analyses were performed on the Linux platform unless mentioned otherwise. Data processing schemes are summarized in Figure S3.

Raw data of read sequences were aligned on the reference genome (Flybase FB2009_09 October, Dmel Release 5.22) using aln, sampe and samse functions (without any options) of BWA software (ver. 0.5.9: http://bio-bwa.sourceforge.net/) [54] and the obtained data (sam files) were converted to the alignment read data (bam file) using the view function of SAMtools software (ver. 0.1.12a: http://samtools.sourceforge.net/) [55]. The bam files were converted to the variant sequence data (pileup files) using pileup functions (with -vcf opsions) of SAMtools. SNPs and InDels (frequency> = 90%, coverage> = 5) were called from pileup files using pileup2snp and pileup2indel functions (with –min-var-freq 0.9 –min-reads2 5 –min-coverage 5 options) of VarScan software (ver. 2.25: http://varscan.sourceforge.net/index.html) [56]. We removed the data on the positions with no information of reference sequence (N in reference) using original bash scripts. Homozygous SNPs and InDels were determined using snpEff software (ver. 1.8, Cingolani, P. “snpEff: Variant effect prediction”, http://snpeff.sourceforge.net, 2011.). Finally, fixed SNPs and InDels were called by filtering the pileup files with the “homozygous” data using compare function of VarScan software. Line-specific and common SNPs between Dark-fly and Oregon-R-S were extracted using the original bash scripts. The distributions of SNPs on chromosomes were analyzed using the “sliding.window” function (http://pbil.univ-lyon1.fr) of the R program developed by The University of Lyon.

### Phylogenetic analysis

The nucleotide sequences of DGRP lines were obtained from the Drosophila Genetic Reference Panel database (http://www.hgsc.bcm.tmc.edu/project-species-i-Drosophila_genRefPanel.hgsc) [21], and sequences of 13 lines were randomly chosen. To minimize the effect of chromosomal regions on the sequence comparison, we carefully selected 8 genes; two genes (chic and drpr) from a region carrying many Dark-fly specific SNPs, two genes (Khc-73 and glec) from a region carrying many Oregon-R-S specific SNPs, two genes (aru and insc) from a region carrying many common SNPs, and two genes (betaInt-nu and tau) from a region with intermingled Dark-fly and Oregon-R-S SNPs (Fig. S1). The sequences of these 8 genes were combined and the combined sequence was used for the phylogenic analysis. The neighbor-joining tree was constructed using the 500 bootstrap test of MEGA (ver.5.05: http://www.megasoftware.net) [57].

### Classification of SNPs and InDels

We used snpEff software to classify SNPs and InDels by their locations relative to gene structures according to the gene annotation data (UCSC dmel 5.22). Our classified groups were intergenic (snpEff terms: intergenic, upstream and downstream), UTR and intron (intron, splice site, UTR 3′, UTR 5′ and start gain), synonymous in coding region (synonymous coding, synonymous start and synonymous end), non-synonymous in coding region (non-synonymous coding, start loss, stop gain and stop loss), InDels in coding region (codon insertion, codon deletion and frameshift) and others (noncoding and unknown). We focused on the non-synonymous SNPs (nsSNPs) and coding InDels (cInDels). Genes carrying nsSNPs and cInDels were classified into Gene Ontology (GO) families (MF4) using the DAVID web-based tool (ver. 6.7: http://david.abcc.ncifcrf.gov/home.jsp) [22], and GO families showing a high probability of gene-enrichment (p-value<0.05) were listed. Nonsense mutations found as “stop gain” by snpEff were confirmed using Integrative Genomics Viewer software (ver. 1.5: http://www.broadinstitute.org/software/igv/home) [58] and current gene annotation data (Flybase, FB2011_07, ver. 5.30).

### Identification and characterization of ROH regions

To obtain data of heterozygous (hetero) and homozygous (homo) SNPs, the BWA-alignment read data (bam files) were converted to the variant call format files (vcf files) using the SAMtools mpileup function (with -B -g –f options) and Bcftools view function (with -c -g -v -N -t 0.1 options). InDel data and low coverage data (less than 5 reads) were removed using the original bash scripts. Vcf files were convert to ped files using Vcftools (ver. 0.1.7: http://vcftools.sourceforge.net/index.html) [59] (with –vcf –plink options). Runs of homozygosity (ROH) regions on major chromosomes (2L, 2R, 3L, 3R and X) were identified using homozyg functions (–homozyg-window-kb 400 –homozyg-kb 200 –homozyg-window-het 2 options) of PLINK software (ver. 1.0.7: http://pngu.mgh.harvard.edu/~purcell/plink/index.shtml) [26].

To evaluate ROH regions under statistical tests, SNPs floating in the population genome (frequency> = 20%) were called from the BWA-alignment read data (bam files) using the SAMtools pileup function (without any options) and VarScan pileup2snp function (with –min-coverage 5 –min-reads2 2 –min-var-freq 0.2 –p-value 0.05 options). SNP frequency (homozygosity) data in the ROH regions were collected using the original bash scripts and were statistically tested by comparing with the average homozygosity of the whole genome using the t.test function (with var.equal = F, alternative = ”greater” options) of R program. Homo SNP fraction of each ROH was also statistically tested by comparing with the average fraction of the whole genome using the fisher.test function (with alternative = ”greater” option) of R program. For graphical analysis, mean homozygosity in the sliding window was calculated from the SNP frequency data using the “sliding.window” function of R program. Mean homozygosity of sliding windows was plotted on chromosomal locations using the R plot function.

### CNV analysis

DNA isolation and purification were done as described in Zhou et al. (2011) [60]. Briefly, genomic DNA was isolated from 60 males, and digested with 1.5 µl of *MspI* restriction enzyme. Restriction digestion followed the manufacturer's recommendations (New England BioLabs) of 37°C for 1 hour. An equal amount of enzyme was added for an additional hour to assure complete digestion. Five micrograms of DNA were used for each sample of Dark-fly and the control, resulting in 10 µg of DNA in each microarray reaction.

Microarrays were ∼18,000-feature cDNA arrays spotted with *D. melanogaster* cDNA PCR products. Labeling and hybridization were conducted using a 3DNA Array 900 MPX kit (Genisphere), with a Cy5-Cy3 two-channel dye swap for each reaction that combines the Dark-fly and control line DNA. After hybridization, microarray slides were scanned in an Axon 4000B scanner (Axon Instruments/Molecular Devices). Scanned microarray slides were first analyzed with GenePix Pro 6.0 software (Axon Instruments/Molecular Devices). Cy5 and Cy3 fluorescence intensities were then normalized by the Loess method in the Limma library of software R (ver. 2.10.1). Bayesian Analysis of Gene Expression Levels (BAGEL) was used to calculate gene copy number increase or decrease relative to the control. BAGEL analysis uses the Bayesian algorithm to compute the probe signal ratios between samples and the reference strain, with p-values indicating the significance (for more details, see [60], [61]). FDRs were estimated based on the variation observed when randomized versions of the original dataset were analyzed. FDRs were smaller than 7%. Array probes located in transposons or containing repetitive sequences were removed from the analyses. The CNV microarray data has been deposited in GEO under accession number GSE35418.

## Supporting Information

Figure S1 **Distribution of SNPs on chromosomes.**
(PDF)

Figure S2 **Phylogenetic tree analysis of the Dark-fly and Oregon-R-S genomes.**
(PDF)

Figure S3 **Schemes of genome data analyses.**
(PDF)

File S1 **File_S1.txt (36 KB).** A tab-separated txt file listed the genes carrying nsSNPs and cInDels in the Dark-fly ROH regions. Chromosome, position, reference nucleotide, altered nucleotide, gene name, reference amino acid/altered amino acid and reference codon/altered condon were presented.
(ZIP)

File S2 **File_S2.txt (8 KB).** A tab-separated txt file listed the genes showing decreased and increased CNVs in the Dark-fly genome. Gene ID, probe ID, p-value and located chromosome were presented.
(TXT)

Table S1 **GO families of genes carrying nsSNPs and cInDels in Dark-fly.**
(PDF)

Table S2 **GO families of genes carrying nsSNPs and cInDels in Oregon-R-S.**
(PDF)

Table S3 **Nonsense mutations in the Dark-fly genome.**
(PDF)

Table S4 **Nonsense mutations in the Oregon-R-S genome.**
(PDF)

Table S5 **ROH regions identified in Dark-fly genome.**
(PDF)

Table S6 **ROH regions identified in Oregon-R-S genome.**
(PDF)

Table S7 **Homozygosity of SNPs in genome.**
(PDF)

Table S8 **Evaluation of the Dark-fly ROH regions.**
(PDF)

Table S9 **GO families of genes carrying nsSNPs and cInDels in the Dark-fly ROH regions.**
(PDF)
