# Supplementary material for: Genome Features of “Dark-Fly”, a Drosophila Line Reared Long-Term in a Dark Environment
Source: PLoS One. 2012 Mar 14;7(3):e33288. doi: 10.1371/journal.pone.0033288 (PMC3303825; doi:10.1371/journal.pone.0033288)
Supplement: Figure S1 — Distribution of SNPs on chromosomes. (PDF) [file pone.0033288.s001.pdf]

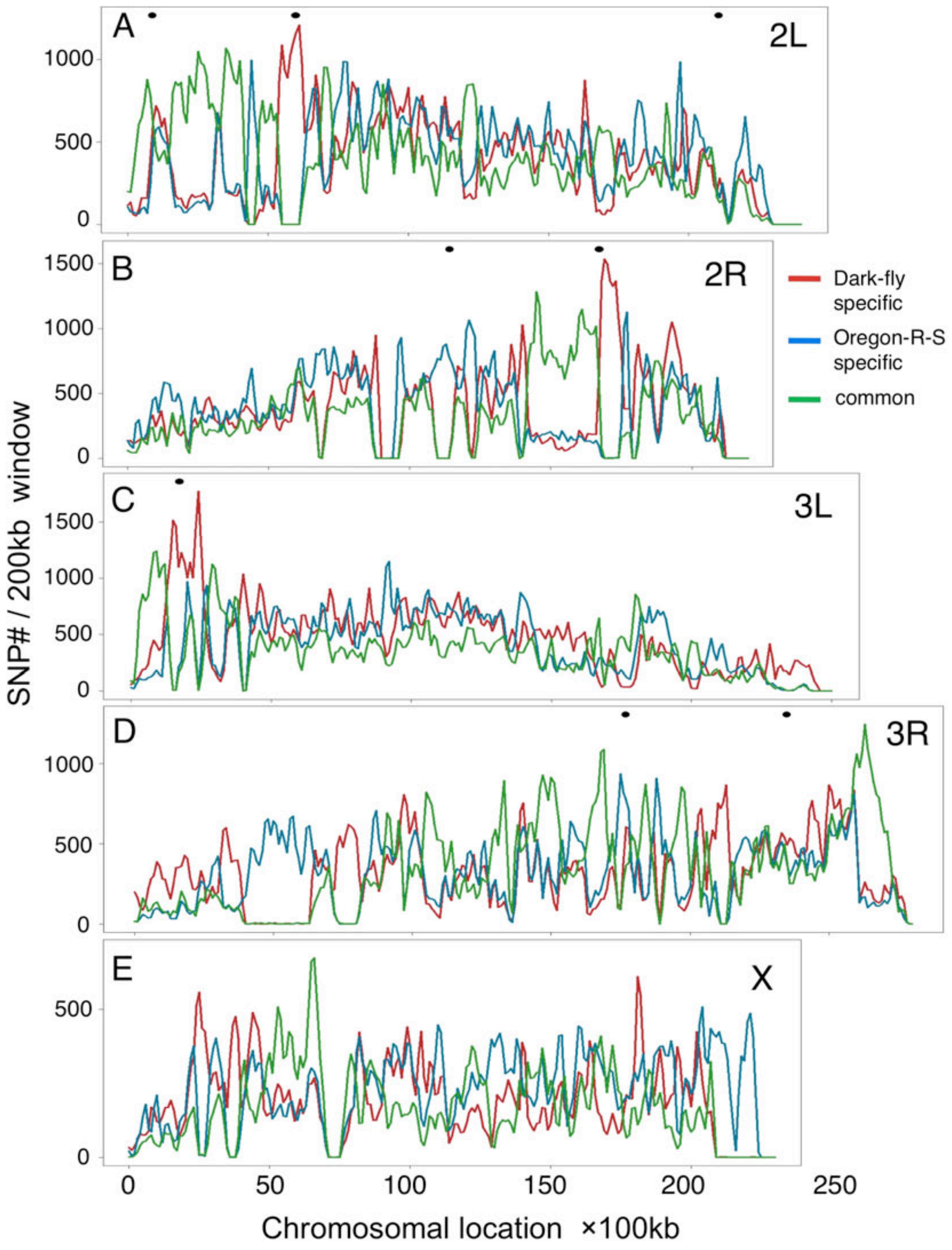

Fig. S1 Distribution of SNPs on chromosomes

Distribution of Dark-fly-specific (red), Oregon-R-S-specific (blue) and common (green) SNPs was analyzed. The number of SNPs in a sliding window (200-kb window at 100-kb steps) was calculated and was plotted versus the locations on 2L (A), 2R (B), 3L (C), 3R (D) and X (E) chromosomes. Location of eight genes used for the phylogenetic analysis (Fig. S2) are indicated by black dots at the top of the graph; aru (2L:861849 (start position)), chic (2L:5972900), betaInt-nu (2L:21052832), Khc-73 (2R:11403283), insc (2R:16708771), drpr (3L:1715594), glec (3R:17676627), and tau (3R:23466512).
