# Supplementary material for: Genome Features of “Dark-Fly”, a Drosophila Line Reared Long-Term in a Dark Environment
Source: PLoS One. 2012 Mar 14;7(3):e33288. doi: 10.1371/journal.pone.0033288 (PMC3303825; doi:10.1371/journal.pone.0033288)
Supplement: Figure S2 — Phylogenetic tree analysis of the Dark-fly and Oregon-R-S genomes. (PDF) [file pone.0033288.s002.pdf]

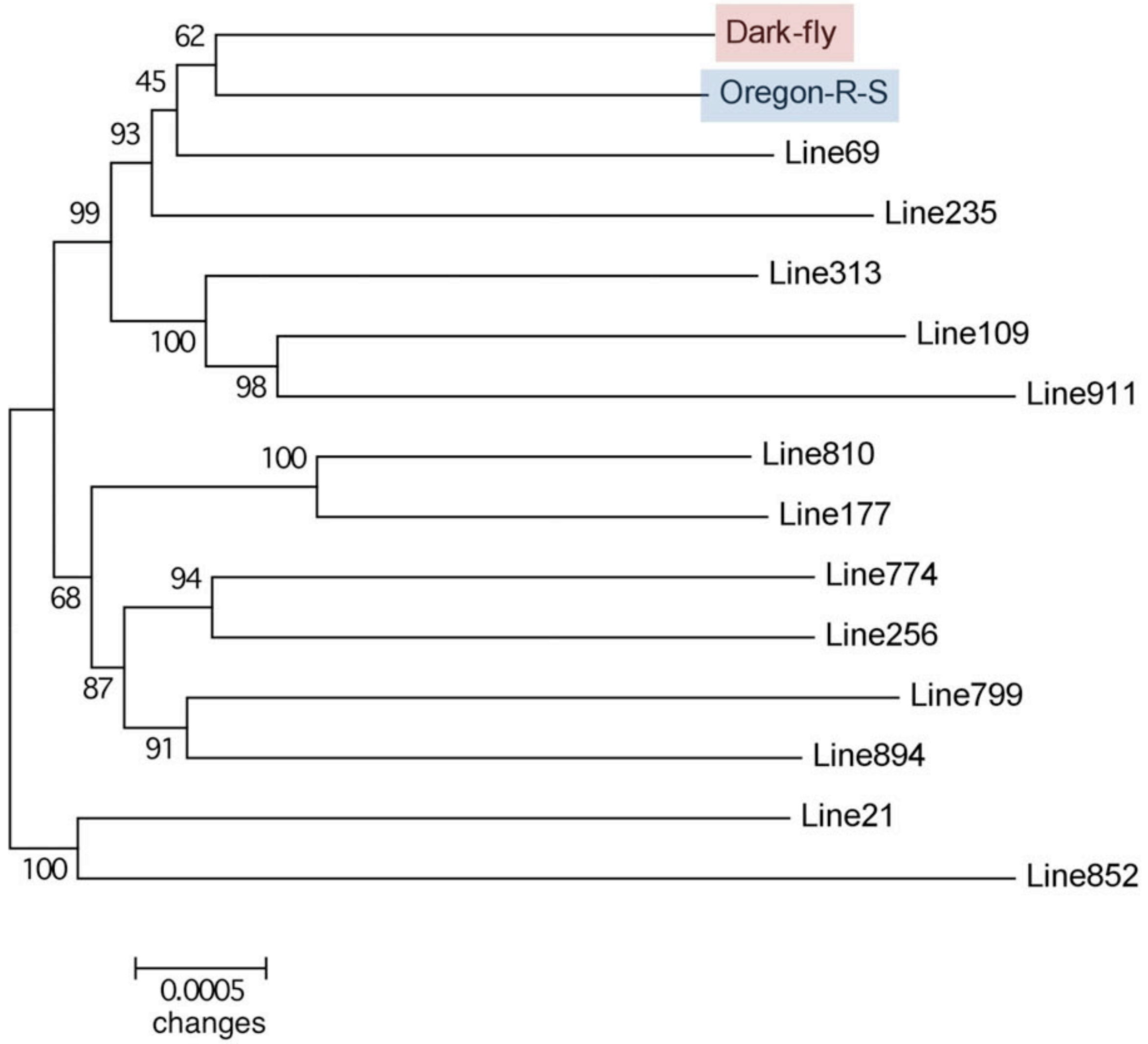

Fig. S2 Phylogenetic tree analysis of the Dark-fly and Oregon-R-S genomes

Neighbor-joining tree with p-distance based on analysis of the combined nucleotide sequence (total 87982 bases) of 8 genes (aru, chic, betaInt-nu, Khc-73, insc, drpr, glec, and tau). The bootstrap value from 500 replications was calculated using MEGA. DGRP lines were randomly selected and are indicated by line number. DGRP lines were highly diverse, whereas Dark-fly (red) and Oregon-R-S (blue) were relatively close on the phylogenetic tree.
