# Supplementary material for: Genome Features of “Dark-Fly”, a Drosophila Line Reared Long-Term in a Dark Environment
Source: PLoS One. 2012 Mar 14;7(3):e33288. doi: 10.1371/journal.pone.0033288 (PMC3303825; doi:10.1371/journal.pone.0033288)
Supplement: Figure S3 — Schemes of genome data analyses. (PDF) [file pone.0033288.s003.pdf]

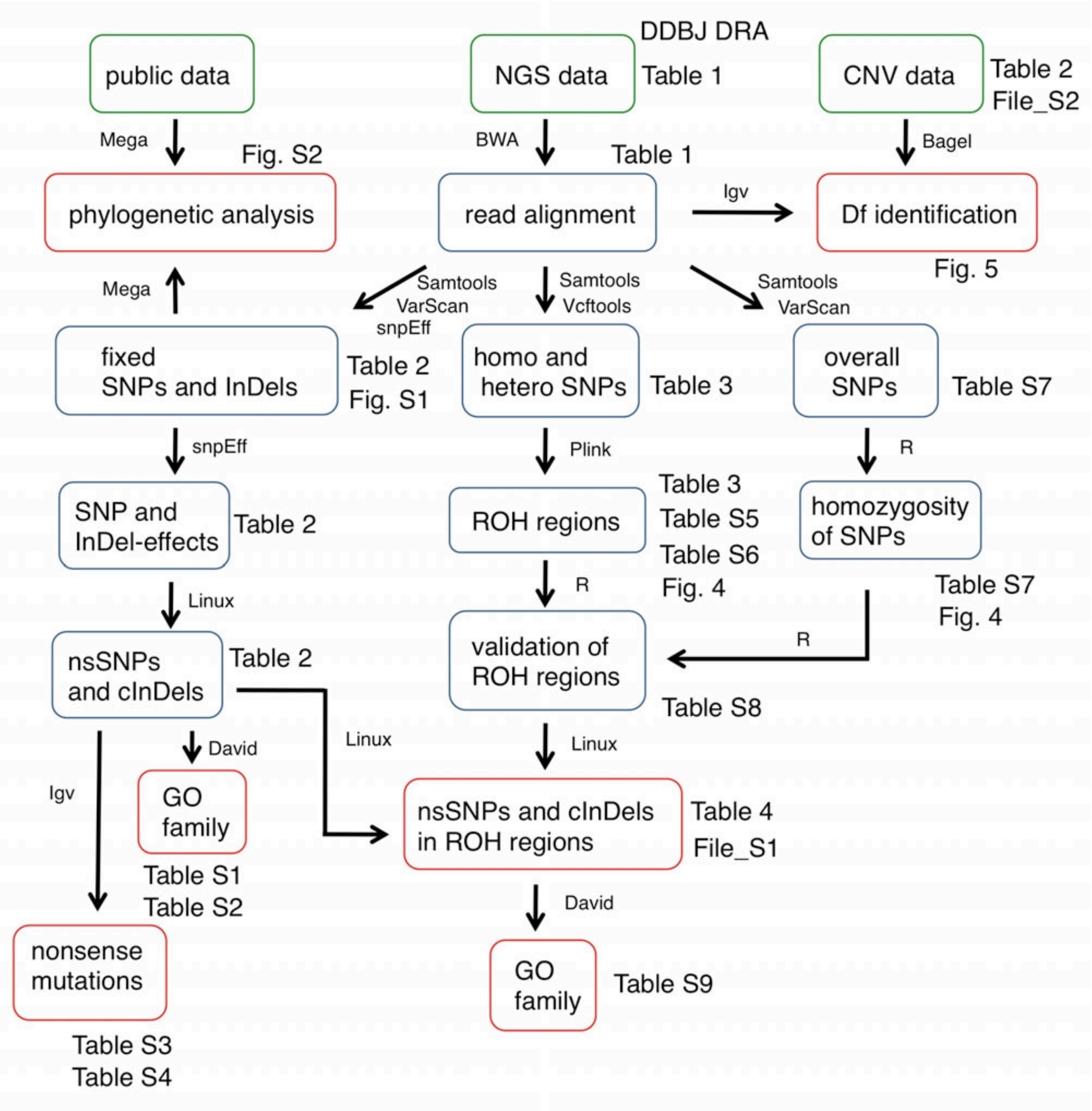

Fig. S3 Schemes of genome data analyses

NGS, CNV and public data (input data: green boxes) were processed through schemes indicated as arrows. Final output data (red boxes) were obtained from intermediate data (blue boxes). Data presented in this report are labeled as figure and table numbers. Softwares used in this study are indicated.
