# Supplementary material for: Genome Features of “Dark-Fly”, a Drosophila Line Reared Long-Term in a Dark Environment
Source: PLoS One. 2012 Mar 14;7(3):e33288. doi: 10.1371/journal.pone.0033288 (PMC3303825; doi:10.1371/journal.pone.0033288)
Supplement: Table S1 — GO families of genes carrying nsSNPs and cInDels in Dark-fly. (PDF) [file pone.0033288.s006.pdf]

Table S1 GO families of genes carrying nsSNPs and cInDels in Dark-fly

GO families (MF4) were listed from the Dark-fly genome data using the DAVID tool (p-value < 0.05). * indicates a family shared with that listed for Oregon-R-S (Table S2). Total gene number annotated in each GO family, gene number counted from data, the Fisher's exact p-value, and fold enrichment of genes are indicated.

GO Term: Molecular function (MF4)<br>*: shared with Oregon-R-S	total gene #	count#	p-value	fold enrich-ment
* GO:0046872~metal ion binding	1718	609	2.40E-06	1.15
* GO:0004888~transmembrane receptor activity	383	159	6.57E-06	1.34
* GO:0008194~UDP-glycosyltransferase activity	92	45	4.56E-04	1.58
GO:0003700~transcription factor activity	389	151	5.63E-04	1.26
GO:0008061~chitin binding	100	47	9.92E-04	1.52
* GO:0016758~transferase activity, transferring hexosyl groups	138	61	1.11E-03	1.43
GO:0004091~carboxylesterase activity	107	47	5.17E-03	1.42
GO:0005085~guanyl-nucleotide exchange factor activity	60	29	7.21E-03	1.56
GO:0043565~sequence-specific DNA binding	230	89	9.33E-03	1.25
GO:0030554~adenyl nucleotide binding	812	281	1.10E-02	1.12
GO:0008528~peptide receptor activity, G-protein coupled	47	23	1.56E-02	1.58
GO:0001653~peptide receptor activity	47	23	1.56E-02	1.58
GO:0008188~neuropeptide receptor activity	45	22	1.86E-02	1.58
GO:0004553~hydrolase activity, hydrolyzing O-glycosyl compounds	100	42	1.98E-02	1.36
GO:0016706~oxidoreductase activity	40	20	2.00E-02	1.62
GO:0015267~channel activity	187	72	2.19E-02	1.25
GO:0004879~ligand-dependent nuclear receptor activity	23	13	2.68E-02	1.83
GO:0004368~glycerol-3-phosphate dehydrogenase activity	7	6	3.26E-02	2.77
GO:0042169~SH2 domain binding	5	5	3.42E-02	3.24
GO:0030594~neurotransmitter receptor activity	70	30	3.93E-02	1.39
