# Supplementary material for: Genome Features of “Dark-Fly”, a Drosophila Line Reared Long-Term in a Dark Environment
Source: PLoS One. 2012 Mar 14;7(3):e33288. doi: 10.1371/journal.pone.0033288 (PMC3303825; doi:10.1371/journal.pone.0033288)
Supplement: Table S2 — GO families of genes carrying nsSNPs and cInDels in Oregon-R-S. (PDF) [file pone.0033288.s007.pdf]

Table S2 GO families of genes carrying nsSNPs and cInDels in Oregon-R-S

Descriptions are the same as for Table S1.

GO Term: Molecular function (MF4)<br>*: shared with Dark-fly	total gene #	count#	p-value	fold enrich-ment
* GO:0004888~transmembrane receptor activity	383	128	1.82E-07	1.51
GO:0004866~endopeptidase inhibitor activity	80	34	8.43E-05	1.92
* GO:0046872~metal ion binding	1718	437	9.37E-05	1.15
GO:0070011~peptidase activity, acting on L-amino acid peptides	612	170	4.27E-04	1.26
GO:0008168~methyltransferase activity	116	41	1.37E-03	1.60
GO:0008559~xenobiotic-transporting ATPase activity	10	8	1.60E-03	3.62
GO:0016646~oxidoreductase activity, acting on the CH-NH group of donors, NAD or NADP as acceptor	14	9	4.58E-03	2.91
GO:0016811~hydrolase activity, acting on carbon-nitrogen (but not peptide) bonds, in linear amides	61	22	1.79E-02	1.63
GO:0004016~adenylate cyclase activity	14	8	1.98E-02	2.59
GO:0015631~tubulin binding	68	23	3.21E-02	1.53
* GO:0016758~transferase activity, transferring hexosyl groups	138	41	3.33E-02	1.35
* GO:0008194~UDP-glycosyltransferase activity	92	29	3.79E-02	1.43
