# Supplementary material for: Genome Features of “Dark-Fly”, a Drosophila Line Reared Long-Term in a Dark Environment
Source: PLoS One. 2012 Mar 14;7(3):e33288. doi: 10.1371/journal.pone.0033288 (PMC3303825; doi:10.1371/journal.pone.0033288)
Supplement: Table S3 — Nonsense mutations in the Dark-fly genome. (PDF) [file pone.0033288.s008.pdf]

Table S3 Nonsense mutations in the Dark-fly genome

Twenty-eight nonsense mutations identified in the Dark-fly genome are listed. Nucleotide position on the chromosome, old nucleotide (in reference genome) and new altered nucleotide (in Dark-fly genome), old and new amino acid, number of affected isoforms / number of total isoforms encoded by gene, and GO term of gene are shown. Ten mutations were located in a subset of gene isoforms (ex. 1/2, 1/3, 6/9 in the "isoforms" column), and 18 mutations altered all products of a gene (ex. 1/1, 2/2, 3/3). * indicates a stop codon.

Chr #	position	old	new	old/new AA	gene name	iso forms	GO: molecular function
2L	3279308	C	T	W/*	CG3332	1/2	-
2L	5710038	G	T	E/*	CG7236	1/2	cyclin-dependent protein kinase activity
2L	6761208	A	T	Y/*	CG11321	1/3	zinc ion binding
2L	9905283	C	A	G/*	CG13124	6/9	-
2L	15291687	G	A	Q/*	CG15260	1/1	-
2L	15671161	G	T	E/*	CG18478	1/1	serine-type endopeptidase activity
2L	16701241	T	A	K/*	CG31782	2/2	nucleic acid binding
2L	19000570	C	G	S/*	CG31792	1/1	transmembrane movement of substances
2L	22443017	T	A	L/*	CG17493	1/1	calcium ion binding
2R	1846832	C	A	Y/*	CG7882	1/1	glucose transmembrane transporter
2R	14184856	C	G	Y/*	CG18538	1/1	-
2R	19724229	C	A	S/*	CG5549	1/1	glycine:sodium symporter activity
3L	2659638	C	A	E/*	CG14950	1/1	-
3L	6321353	C	T	Q/*	Or65c	1/1	olfactory receptor activity
3L	7754660	C	T	Q/*	Hn	1/2	phenylalanine 4-monooxygenase activity
3L	8505556	G	T	E/*	CG6745	1/1	pseudouridine synthase activity
3L	8512983	C	T	Q/*	CG6776	1/1	glutathione transferase activity
3L	10883372	C	T	Q/*	Cpr67Fa1	1/1	chitin-based larval cuticle
3L	12159147	C	A	E/*	Rh7	1/1	G-protein coupled photoreceptor activity
3L	12958251	G	A	Q/*	Or69a	1/2	olfactory receptor activity
3R	145542	G	T	E/*	CG9795	1/4	-
3R	7614125	C	T	R/*	HisCl1	1/4	histamine-gated chloride channel
3R	14079539	C	A	E/*	Rpb4	2/6	histone acetyltransferase activity
3R	19422977	G	C	S/*	CG10183	1/1	transferring acyl groups
3R	20666275	C	A	E/*	CG33658	1/1	-
3R	21511115	T	A	K/*	CG4774	3/3	CDP-alcohol phosphatidyltransferase
3R	24956962	G	A	W/*	Vha100-1	1/10	hydrogen-exporting ATPase activity
X	2222878	G	T	E/*	CG2854	1/1	-
