# Supplementary material for: Genome Features of “Dark-Fly”, a Drosophila Line Reared Long-Term in a Dark Environment
Source: PLoS One. 2012 Mar 14;7(3):e33288. doi: 10.1371/journal.pone.0033288 (PMC3303825; doi:10.1371/journal.pone.0033288)
Supplement: Table S4 — Nonsense mutations in the Oregon-R-S genome. (PDF) [file pone.0033288.s009.pdf]

Table S4 Nonsense mutations in the Oregon-R-S genome

Twenty-three nonsense mutations identified in the Oregon-R-S genome are listed. Nucleotide position on the chromosome, old nucleotide (in reference genome) and new altered nucleotide (in Oregon-R-S genome), old and new amino acid, and GO term of gene are shown. * indicates a stop codon.

| Chr # | position | old | new | old/new AA | gene name | GO: molecular function |
|---|---|---|---|---|---|---|
| 2L | 8961925 | C | A | E/* | CG9525 | - |
| 2L | 10820205 | G | A | W/* | CG6508 | aspartic-type endopeptidase activity |
| 2L | 18356266 | G | A | Q/* | Acp36DE | hormone activity |
| 2L | 19183181 | A | T | C/* | CG17567 | - |
| 2L | 19559469 | A | T | R/* | CG13079 | endopeptidase inhibitor activity |
| 2L | 19559877 | C | T | Q/* | CG13079 | endopeptidase inhibitor activity |
| 2R | 5310836 | C | A | E/* | CG13955 | - |
| 2R | 6009161 | T | A | K/* | CCS | superoxide dismutase copper chaperone activity |
| 2R | 6166951 | C | T | Q/* | CAP | vinculin binding |
| 2R | 10626740 | C | T | Q/* | CG12865 | - |
| 2R | 10657481 | C | T | W/* | CG42254 | - |
| 2R | 10705467 | G | A | Q/* | CG10202 | phosphomannomutase activity |
| 2R | 19964884 | C | A | Y/* | Mlp60A | zinc ion binding |
| 2R | 20184781 | C | G | Y/* | CG3394 | long-chain fatty acid transporter activity |
| 3L | 6063062 | C | A | S/* | CG42269 | secondary active organic cation transmembrane transporter activity |
| 3R | 6484581 | A | T | K/* | CG34304 | - |
| 3R | 6953442 | A | T | L/* | Ugt86Dd | glucuronosyltransferase activity |
| 3R | 8817309 | C | T | W/* | yellow-f | dopachrome isomerase activity |
| 3R | 18925930 | G | A | W/* | CG6738 | aminoacylase activity |
| 3R | 20386246 | G | T | S/* | CG13624 | protein homodimerization activity |
| 3R | 21728852 | G | T | G/* | CG31089 | triglyceride lipase activity |
| X | 11306744 | G | C | Y/* | Gr10b | taste receptor activity |
| X | 15963716 | C | T | R/* | dpr18 | - |
