# Supplementary material for: Genome Features of “Dark-Fly”, a Drosophila Line Reared Long-Term in a Dark Environment
Source: PLoS One. 2012 Mar 14;7(3):e33288. doi: 10.1371/journal.pone.0033288 (PMC3303825; doi:10.1371/journal.pone.0033288)
Supplement: Table S5 — ROH regions identified in Dark-fly genome. (PDF) [file pone.0033288.s010.pdf]

Table S5 ROH regions identified in Dark-fly genome

The chromosomal position and length of the Dark-fly ROH regions are showed. Number of SNPs and homo SNP fraction in each ROH region were derived from the PLINK data.

ROH ID	Chr	position start	position end	length bases	# of SNP	homo SNP fraction %
ROH1	2L	3353705	3669168	315463	2103	98.7
ROH2	2L	6535198	6782752	247554	1411	98.3
ROH3	2L	8847085	9109796	262711	1549	98.2
ROH4	2L	10278630	10524864	246234	1393	98.6
ROH5	2L	13521459	13806482	285023	1287	97.7
ROH6	2L	13806743	14034237	227494	1153	97.7
ROH7	2L	15628469	15854613	226144	905	97.9
ROH8	2R	2174959	2453604	278645	366	95.9
ROH9	2R	2722221	2975600	253379	694	96.7
ROH10	2R	12738094	13006423	268329	1152	97.8
ROH11	3L	3118085	3327625	209540	1007	98.3
ROH12	3L	7532105	7813157	281052	1447	97.9
ROH13	3L	14059399	14275678	216279	1263	97.6
ROH14	3L	15737620	15945049	207429	667	97.0
ROH15	3L	18793182	19024297	231115	858	97.4
ROH16	3L	20560665	20819130	258465	404	96.0
ROH17	3L	22471441	22725139	253698	510	97.6
ROH18	3R	80650	293579	212929	113	94.7
ROH19	3R	2862778	3085343	222565	445	97.3
ROH20	3R	3257401	3475620	218219	812	97.0
ROH21	3R	8358059	8659641	301582	1009	97.6
ROH22	3R	9912039	10141059	229020	1183	98.3
ROH23	3R	12540659	12771162	230503	866	97.7
ROH24	3R	22056540	22307403	250863	947	97.8
