# Supplementary material for: Genome Features of “Dark-Fly”, a Drosophila Line Reared Long-Term in a Dark Environment
Source: PLoS One. 2012 Mar 14;7(3):e33288. doi: 10.1371/journal.pone.0033288 (PMC3303825; doi:10.1371/journal.pone.0033288)
Supplement: Table S6 — ROH regions identified in Oregon-R-S genome. (PDF) [file pone.0033288.s011.pdf]

Table S6 ROH regions identified in Oregon-R-S genome

The chromosomal position and length of the Oregon-R-S ROH regions are showed. Number of SNPs and homo SNP fraction in each ROH region were derived from the PLINK data.

ROH ID	Chr	position start	position end	length bases	# of SNP	homo SNP fraction %
ROH1	2L	165238	750090	584852	1766	98.2
ROH2	2L	1258757	1661259	402502	1976	98.0
ROH3	2L	1819818	2096126	276308	1526	98.0
ROH4	2L	2096255	2315836	219581	849	99.2
ROH5	2L	2374388	2606881	232493	1235	98.4
ROH6	2L	3266835	4028337	761502	4950	98.5
ROH7	2L	4028708	4236733	208025	1361	98.2
ROH8	2L	4913174	5318480	405306	1789	98.8
ROH9	2L	6377338	6950235	572897	3135	98.6
ROH10	2L	7313250	8081683	768433	5094	98.9
ROH11	2L	8432337	9051929	619592	3425	98.4
ROH12	2L	9389894	9597448	207554	1163	97.6
ROH13	2L	9828401	10771950	943549	5843	98.6
ROH14	2L	12118599	12579022	460423	2945	98.3
ROH15	2L	13273633	13574305	300672	1078	99.0
ROH16	2L	14237037	14455195	218158	1171	98.2
ROH17	2L	14455643	14744809	289166	1187	98.6
ROH18	2L	15254596	15505570	250974	1043	98.8
ROH19	2L	15506651	16191292	684641	2955	98.8
ROH20	2L	16265368	16504424	239056	1282	98.4
ROH21	2L	17021969	17244822	222853	916	98.9
ROH22	2L	17578245	17784812	206567	765	97.5
ROH23	2L	18044413	18444778	400365	1717	98.5
ROH24	2L	18542931	19077948	535017	2108	98.1
ROH25	2L	19265759	19591515	325756	1327	98.6
ROH26	2L	20604627	20904961	300334	1381	98.0
ROH27	2L	21037950	21386042	348092	702	97.6
ROH28	2R	1714190	1977974	263784	839	98.6
ROH29	2R	2401639	2655392	253753	871	97.7
ROH30	2R	2656600	2913395	256795	792	98.2
ROH31	2R	3083913	3306073	222160	617	98.5
ROH32	2R	3956668	4216764	260096	932	97.4
ROH33	2R	4219148	4926591	707443	2598	97.7
ROH34	2R	4927135	5206138	279003	1008	98.2
ROH35	2R	5605491	6011267	405776	1828	98.4
ROH36	2R	6106349	6313956	207607	1609	98.9
ROH37	2R	6959669	7253563	293894	1423	98.0
ROH38	2R	8143904	8504774	360870	2057	98.6
ROH39	2R	8505822	8815736	309914	1426	98.3
ROH40	2R	9597872	9905341	307469	1603	98.6

ROH41	2R	10332041	10636882	304841	1927	99.0
ROH42	2R	10670548	10887475	216927	1208	98.3
ROH43	2R	11974558	12253239	278681	1767	98.1
ROH44	2R	12339888	12856716	516828	2549	98.1
ROH45	2R	13022838	13671883	649045	3153	98.2
ROH46	2R	13681250	13908451	227201	1263	98.3
ROH47	2R	14237195	14776816	539621	3484	98.7
ROH48	2R	14779403	15176269	396866	2168	98.2
ROH49	2R	15381022	15592697	211675	1158	97.2
ROH50	2R	15593338	15820228	226890	988	98.6
ROH51	2R	15821115	16082871	261756	1338	98.2
ROH52	2R	17657315	17880999	223684	1666	98.6
ROH53	2R	18925977	19309771	383794	2036	97.6
ROH54	2R	19680837	20057676	376839	1642	98.7
ROH55	2R	20774480	20981117	206637	537	98.5
ROH56	3L	458770	725179	266409	1323	97.7
ROH57	3L	2835096	3051163	216067	1795	98.8
ROH58	3L	3116845	3403928	287083	1369	98.2
ROH59	3L	3640122	3853685	213563	1651	98.5
ROH60	3L	4615408	4924686	309278	1844	98.2
ROH61	3L	4961996	5186023	224027	1277	98.4
ROH62	3L	5845082	6433003	587921	2622	98.2
ROH63	3L	6635265	6874367	239102	1484	98.0
ROH64	3L	6988623	7332133	343510	1777	98.3
ROH65	3L	7357846	7762925	405079	1918	98.7
ROH66	3L	7870841	8321646	450805	2117	98.5
ROH67	3L	8816309	9069670	253361	1262	98.6
ROH68	3L	9515287	9874786	359499	2257	98.2
ROH69	3L	10101180	10519050	417870	2871	98.1
ROH70	3L	11571868	11850061	278193	1706	99.0
ROH71	3L	11956952	12535529	578577	3801	98.4
ROH72	3L	12651606	13067659	416053	2271	98.2
ROH73	3L	13096570	13577508	480938	2700	98.4
ROH74	3L	13918590	14430926	512336	3248	98.5
ROH75	3L	17602063	17820364	218301	775	99.0
ROH76	3L	17869631	18220463	350832	1784	98.6
ROH77	3L	18220844	18426241	205397	1516	99.0
ROH78	3L	18426531	18791996	365465	1820	98.0
ROH79	3L	18815536	19095180	279644	1504	98.3
ROH80	3L	19839488	20164581	325093	791	97.2
ROH81	3L	21150093	21357593	207500	478	97.9
ROH82	3L	21570556	22140802	570246	1015	97.9
ROH83	3L	22307180	22537945	230765	529	97.9
ROH84	3L	22546928	22821926	274998	424	97.6
ROH85	3R	2437525	2774309	336784	814	98.0
ROH86	3R	2781878	3005407	223529	598	97.3
ROH87	3R	4699779	5005569	305790	996	97.9
ROH88	3R	5013641	5273825	260184	859	97.6

ROH89	3R	5476101	5687531	211430	520	98.1
ROH90	3R	5892947	6306641	413694	900	98.0
ROH91	3R	6509058	6989361	480303	1334	97.8
ROH92	3R	8539383	8763152	223769	998	97.8
ROH93	3R	9110548	9313555	203007	1226	98.6
ROH94	3R	9816859	10124148	307289	987	98.9
ROH95	3R	10124319	10533993	409674	1959	98.1
ROH96	3R	10534351	10957104	422753	1998	98.3
ROH97	3R	10961237	11237632	276395	976	98.3
ROH98	3R	11305503	11622233	316730	1054	98.3
ROH99	3R	12041092	12408360	367268	1260	98.2
ROH100	3R	12411027	13203990	792963	2820	98.2
ROH101	3R	13382188	13621438	239250	778	97.9
ROH102	3R	14022564	14246294	223730	1317	98.3
ROH103	3R	14593470	15592540	999070	5834	98.1
ROH104	3R	15718619	15940748	222129	1087	98.3
ROH105	3R	15992189	16285510	293321	1465	98.3
ROH106	3R	16287343	16627103	339760	1421	99.1
ROH107	3R	16770503	17022175	251672	1662	98.6
ROH108	3R	17202886	17557516	354630	1760	97.9
ROH109	3R	17788454	18002227	213773	1244	98.5
ROH110	3R	18496399	18783764	287365	1446	97.7
ROH111	3R	19670298	20031137	360839	1805	98.5
ROH112	3R	20055572	20321387	265815	1056	97.9
ROH113	3R	21684800	22025589	340789	1212	98.3
ROH114	3R	22211351	22450505	239154	1108	98.3
ROH115	3R	22671104	22956148	285044	1760	99.0
ROH116	3R	22956440	23158144	201704	810	98.1
ROH117	3R	23469327	23685882	216555	1043	97.9
ROH118	3R	23826115	24117008	290893	1197	98.3
ROH119	3R	25488537	25796103	307566	2162	98.0
ROH120	3R	25864277	26127250	262973	2257	99.0
ROH121	3R	26797316	27151156	353840	1531	98.8
ROH122	3R	27608563	27897728	289165	207	95.2
ROH123	X	2212719	2421411	208692	621	97.3
ROH124	X	12200095	12424481	224386	856	97.3
ROH125	X	14810300	15031589	221289	663	97.4
ROH126	X	15226498	15428641	202143	516	96.9
ROH127	X	16869249	17278078	408829	1474	97.6
ROH128	X	20330504	20540461	209957	770	97.3
