# Supplementary material for: Genome Features of “Dark-Fly”, a Drosophila Line Reared Long-Term in a Dark Environment
Source: PLoS One. 2012 Mar 14;7(3):e33288. doi: 10.1371/journal.pone.0033288 (PMC3303825; doi:10.1371/journal.pone.0033288)
Supplement: Table S7 — Homozygosity of SNPs in genome. (PDF) [file pone.0033288.s012.pdf]

Table S7 Homozygosity of SNPs in genome

Overall SNPs (0.2 =< frequency) were indentified using VarScan program and were classified into homo SNPs (0.9 =< frequency) and floating SNPs (0.2 =< frequency < 0.9). The number of homo SNPs was slightly different from that of the homo SNPs in Table 2 and Table 3, due to the difference of data filtering. Homo SNP fraction and mean SNP frequency (homozygosity) were calculated from the VarScan data. * indicates significant difference of mean homozygosity between Dark-fly and Oregon-R-S (Welch t-test, p-value = 2.2E-16).

fly line	Dark-fly	Oregon-R-S
overall SNPs (0.2 =< freq)	522,391	499,507
homo SNPs (0.9 =< freq)	463,633	445,666
floating SNPs (0.2 =< freq < 0.9)	58,758	53,841
homo SNP fraction (%)	88.8	89.2
homozygosity (mean±SD) *	0.941±0.176	0.944±0.171
