# Supplementary material for: Genome Features of “Dark-Fly”, a Drosophila Line Reared Long-Term in a Dark Environment
Source: PLoS One. 2012 Mar 14;7(3):e33288. doi: 10.1371/journal.pone.0033288 (PMC3303825; doi:10.1371/journal.pone.0033288)
Supplement: Table S8 — Evaluation of the Dark-fly ROH regions. (PDF) [file pone.0033288.s013.pdf]

Table S8 Evaluation of the Dark-fly ROH regions

Numbers of overall SNPs and homo SNP fraction in each ROH region were calculated from the VarScan data, and mean homozygosity were calculated using the R program. Statistical tests were performed by comparing mean homozygosity of each ROH region with mean homozygosity of the whole genome using the Welch t-test and by comparing homo SNP fraction of each ROH region with homo SNP fraction of the whole genome using the Fisher's exact test. Three of 24 ROH regions (ROH ID#8, 12 and 18) failed to show significantly high homozygosity (p-value < 0.01).

ROH ID#	Ch.	position start	position end	SNP #	homo SNP fraction %	homozygosity mean±SD	p-value Welch t-test	p-value Fisher's test
ROH1	2L	3353705	3669168	2244	95.4	0.976 ± 0.116	< 2.2e-16	< 2.2e-16
ROH2	2L	6535198	6782752	1496	96.5	0.983 ± 0.096	< 2.2e-16	< 2.2e-16
ROH3	2L	8847085	9109796	1647	96.5	0.984 ± 0.093	< 2.2e-16	< 2.2e-16
ROH4	2L	10278630	10524864	1477	97.5	0.988 ± 0.078	< 2.2e-16	< 2.2e-16
ROH5	2L	13521459	13806482	1420	94.1	0.980 ± 0.125	< 2.2e-16	3.61E-12
ROH6	2L	13806743	14034237	1223	96.3	0.971 ± 0.107	< 2.2e-16	< 2.2e-16
ROH7	2L	15628469	15854613	1006	96.5	0.982 ± 0.102	< 2.2e-16	< 2.2e-16
ROH8	2R	2174959	2453604	443	74.7	0.865 ± 0.252	1	1
ROH9	2R	2722221	2975600	741	94.7	0.972 ± 0.122	4.47E-12	1.09E-08
ROH10	2R	12738094	13006423	1247	94.7	0.974 ± 0.119	< 2.2e-16	1.87E-13
ROH11	3L	3118085	3327625	1094	95.7	0.978 ± 0.110	< 2.2e-16	< 2.2e-16
ROH12	3L	7532105	7813157	1637	90.2	0.948 ± 0.169	4.28E-02	3.06E-02
ROH13	3L	14059399	14275678	1346	96.1	0.981 ± 0.100	< 2.2e-16	< 2.2e-16
ROH14	3L	15737620	15945049	765	92.4	0.963 ± 0.141	9.71E-06	4.73E-04
ROH15	3L	18793182	19024297	923	96.0	0.983 ± 0.092	< 2.2e-16	2.64E-15
ROH16	3L	20560665	20819130	436	93.1	0.963 ± 0.146	1.04E-03	1.46E-03
ROH17	3L	22471441	22725139	545	95.8	0.978 ± 0.112	3.25E-14	4.27E-09
ROH18	3R	80650	293579	123	90.2	0.941 ± 0.180	5.12E-01	3.64E-01
ROH19	3R	2862778	3085343	468	94.9	0.968 ± 0.144	3.73E-05	3.05E-06
ROH20	3R	3257401	3475620	874	95.4	0.973 ± 0.126	1.95E-13	2.49E-12
ROH21	3R	8358059	8659641	1099	94.7	0.971 ± 0.127	5.77E-15	4.19E-12
ROH22	3R	9912039	10141059	1272	95.7	0.978 ± 0.113	< 2.2e-16	< 2.2e-16
ROH23	3R	12540659	12771162	939	94.8	0.971 ± 0.129	1.47E-12	9.12E-11
ROH24	3R	22056540	22307403	1019	96.0	0.981 ± 0.100	< 2.2e-16	< 2.2e-16
