# Supplementary material for: Genome Features of “Dark-Fly”, a Drosophila Line Reared Long-Term in a Dark Environment
Source: PLoS One. 2012 Mar 14;7(3):e33288. doi: 10.1371/journal.pone.0033288 (PMC3303825; doi:10.1371/journal.pone.0033288)
Supplement: Table S9 — GO families of genes carrying nsSNPs and cInDels in the Dark-fly ROH regions. (PDF) [file pone.0033288.s014.pdf]

Table S9 GO families of genes carrying nsSNPs and cInDels in the Dark-fly ROH regions

GO families (MF4) were listed from the data of genes in the Dark-fly ROH regions using the DAVID tool. Descriptions are the same as for Table S1.

| GO Term: Molecular function (MF4) | total gene # | count# | p-value | fold enrich-ment |
|---|---|---|---|---|
| GO:0004091~carboxylesterase activity | 107 | 10 | 8.89E-05 | 5.36 |
| GO:0005083~small GTPase regulator activity | 108 | 7 | 1.08E-02 | 3.72 |
| GO:0005085~guanyl-nucleotide exchange factor activity | 60 | 5 | 1.97E-02 | 4.78 |
